# Influence of an External Electric Field and Dissipative Tunneling on Recombination Radiation in Quantum Dots

**DOI:** 10.3390/s22041300

**Published:** 2022-02-09

**Authors:** Vladimir D. Krevchik, Aleksei V. Razumov, Mikhail B. Semenov, Saygid U. Uvaysov, Vladimir P. Kulagin, Paweł Komada, Saule Smailova, Aisha Mussabekova

**Affiliations:** 1 Faculty of Information Technology and Electronics, Penza State University, 440026 Penza, Russia; fpite@pnzgu.ru (V.D.K.); physics@pnzgu.ru (A.V.R.); 2 MIREA—Russian Technological University, 119454 Moscow, Russia; uvajsov@mirea.ru (S.U.U.); kulagin@mirea.ru (V.P.K.); 3 Department of Electronics and Information Technologies, Lublin University of Technology, Nadbystrzycka 38d, 20-618 Lublin, Poland; p.komada@pollub.pl; 4 D. Serikbayev East Kazakhstan Technical University, 69 Protozanov Str., Ust-Kamenogorsk 070004, Kazakhstan; saule_smailova@mail.ru; 5 Academy of Logistics and Transport, 97 Shevchenko Str., Almaty 050012, Kazakhstan; aisha_1292@mail.ru

**Keywords:** dissipative tunneling, spectral intensity of recombination radiation, quasi-stationary *A^+^*-state, quantum dot, impurity complexes

## Abstract

The effect of an external electric field and dissipative tunneling on the spectral intensity of recombination radiation in a quantum dot with an *A^+^ + e* impurity complex (a hole localized on a neutral acceptor interacting with an electron localized in the ground state of the quantum dot) is studied in the zero-radius potential model in the adiabatic approximation. The probability of dissipative tunneling of a hole is calculated in the one-instanton approximation. A high sensitivity of the recombination radiation intensity to the strength of the external electric field and to such parameters of the surrounding matrix (dissipative tunneling parameters) as temperature, the constant of interaction with the contact medium (or the heat-bath), and the frequency of phonon modes, has been revealed. It is shown that an external electric field leads to a shift of the recombination radiation threshold by several tens of meV, and a change in the parameters of dissipative tunneling has a noticeable effect on the spectral intensity of recombination radiation. It is shown that the resonant tunneling effect manifests itself in the form of “dips” in the field dependence of the spectral intensity of recombination radiation, which occur at certain values of the external electric field strength and temperature. This opens up certain prospects for the use of the considered system “quantum dot—impurity complex *A^+^ + e*” under conditions of dissipative tunneling for the study and diagnostics of biological objects.

## 1. Introduction

As is known, quantum technologies are currently a priority direction in the development of sensors. In particular, there are three groups of quantum sensors: “(1) clocks, gravimeters, gradiometers; (2) electric and magnetic field sensors; (3) sensors for quantum metrology”. It was noted that “… the IPS SB RAS has begun the development of single photon detectors based on avalanche photodiodes with heterostructures for fiber-optic quantum communications; at Moscow State University named by M.V. Lomonosov, in the framework of the direction of quantum metrology of the roadmap for quantum sensors, physical foundations have been developed and prototypes of devices for “absolute quantum photometry” have been built…; at the Physico-technical Institute named by A.F. Ioffe, RAS, the fundamental problem of detecting weak magnetic fields with nm—resolution has been solved. … Nanocrystals of a given polytype have been created, which can be combined with confocal and probe microscopy, resulting in atomic spatial resolution”. Research in this field, in particular, based on quantum dots with impurity complexes in the external control electric field, is undoubtedly a topical area of research in the field of modern optoelectronics. An important factor that significantly determines the optical response of quantum dots, in addition to the external electric field, is the influence of the surrounding matrix within the framework of the dissipative tunneling mechanism [1,2,3,4]. When discussing the effect of the surface on the photoluminescence of semiconductor quantum dots during the development of quantum sensors, it was noted [5] that it is important to take into account as role of recombination radiation as also tunneling mechanism. Among the possible mechanisms of the quantum dots interaction with the environment (with the heat-bath), we propose to take into account the mechanism of quantum tunneling with dissipation [1,2,3,4]. In addition, as model parameters of dissipative tunneling, it is necessary to take into account the temperature of the matrix surrounding the quantum dot with the impurity complex “*A^+^ + e*”) in the external control electric field, the parameter of interaction with the contact medium (with the heat-bath), frequencies of optical and acoustic phonon modes of the heat-bath matrix.

At present, interest is attracted by the effect of recombination radiation in the development of quantum sensors [5]. 

It is well known [6] that the core-shell quantum dots have a higher quantum yield of radiation than fluorescent chromophores, optical activity in the long-wavelength region of the spectrum, and significantly higher photochemical stability. Thus, these spectral properties of the core-shell quantum dots are very promising for research in biology and medicine and for the usage of the core-shell quantum dots as biological sensors. In [6], a new method for diagnosing amino acids (or other ligands) using quantum dots was proposed. We are talking about the effect of the interaction of amino acids (ligands) with quantum dots on the energy spectrum of quantum dots. This interaction affects the spectrum of radiative recombination of electrons and holes in quantum dots due to the different distribution of the field around them. Therefore, by changing the spectrum of radiative recombination of the core-shell quantum dots, it is possible to identify a biological object. 

There are well-known works where the usage of quantum dots as biomedical sensors has been investigated ([6,7]; see also the author’s patent [8]; [9,10]). Considering our work on the “Influence of an external electric field and dissipative tunneling on recombination radiation in quantum dots”, we also assume the usage of the proposed model for the purposes of modern nanomedicine and optoelectronics with controllable characteristics. 

**Figure 1:**
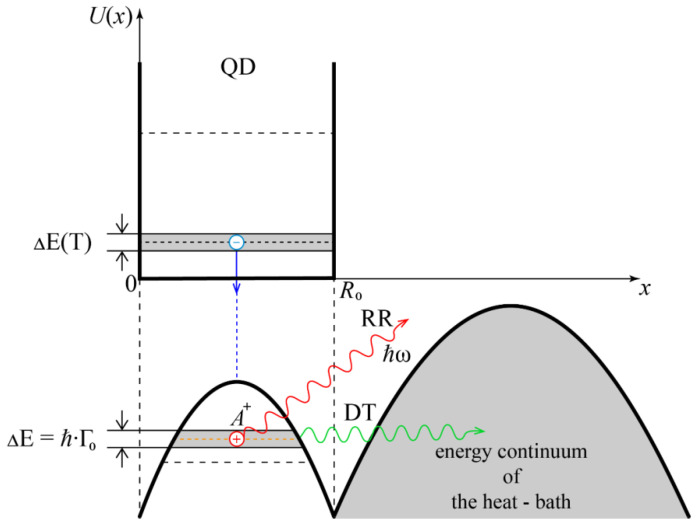
Energy structure of considered system based on the “quantum dot—impurity complex *A^+^ + e*” in the presence of dissipative tunneling. The electron is localized in the ground state of the quantum dot, and the hole is in the quasi-stationary state of the *A^+^*-center: ΔE(T)- is the magnitude of the temperature broadening of the QD electron energy level; ΔE = *ħ*Γ_0_—is the broadening of the impurity level associated with the tunneling decay of the *A*^+^-state; RR—recombination radiation; DT—dissipative tunneling.

The aim of this work is to show that the recombination radiation in the “quantum dot—impurity complex” system under the conditions of dissipative tunneling can be effectively used to determine parameters of the medium surrounding the quantum dot. This is important, for example, for nanomedicine, where the diagnosis of amino acids [6,7] and of oncological tumors [8] takes place, as well as for nanotechnology of quasi-zero-dimensional structures in cases where the surrounding matrix or the heat-bath can lead to a significant modification of the energy spectrum of the array of quantum dots due to tunneling transparency of potential barriers. Figure 1 shows the structure of the considered system “quantum dot—impurity complex”. 

Note that most of the currently existing works that take into account the tunneling mechanism in describing the optical properties of quantum dots offer only numerical estimates of the results obtained. One of the main advantages of this work is obtaining the main results in an analytical form. 

## 2. Materials and Methods

The theoretical consideration of the temperature effect on the energy levels in a semiconductor quantum dot (QD) was carried out by a statistical method, under assumption that the main contribution to the temperature dependence is made by the electron-phonon interaction. The dispersion equation, which determines the binding energy of a hole in an impurity complex $A^{+}+e$ in a spherically symmetric quantum dot, has been obtained in framework of the adiabatic approximation by the zero-range potential method. Calculation of the dissipative tunneling probability is performed in the one-instanton approximation. Calculation of the recombination radiation intensity in a quasi-zero-dimensional structure with impurity complexes is performed in the dipole approximation taking into account the radius dispersion of quantum dots and the Lorentz broadening of energy levels. The curves are plotted for the case of InSb quantum dots. In semiconductor nanostructures, the concept of deep and shallow impurities is relative since the depth of the impurity level depends on the characteristic size of the nanostructure. *A^+^*-centers appear due to the attachment of an additional hole to a neutral acceptor, and the interaction potential of a hole with a neutral acceptor is not Coulomb, but short-range. Such centers have been found in quantum wells (GaAs/AlGaAs) [11,12]. As is known, the effective mass approximation is applicable if the exciton Bohr radius $a_{ex}$ is large compared to the crystal lattice constant a. For a QD with a radius $R_{0}$, the applicability condition for the effective mass method is that it ($R_{0}$) must exceed the a value by several orders of magnitude. It is easy to show that this criterion is satisfied by semiconductors with a small effective electron mass. Thus, for *InSb*-based QDs with effective masses of electrons $m_{e}^{*}=0.013m_{e}$ and holes $m_{h}^{*}=0.6m_{e}$, the exciton Bohr radius is $a_{ex}\approx 70\;\mathrm{nm}$, which is two orders of magnitude larger than the lattice constant (a=0.65 nm). In this work, calculations and plotting are performed for the radius of the QD, in this case $N=\frac{R_{0}}{a}=108$. Thus, the number of atomic layers in an *InSb* crystal turns out to be sufficient for the applicability of the effective mass method. The value of *N* given can serve as an estimate for the number of unit cells of the material crystals, which are contained in the QD.

## 3. Results

### 3.1. Binding Energy of a Quasi-Stationary A^+^-State in a Quantum Dot in the Presence of Tunneling Decay in an External Electric Field

Let us consider *A^+^*-center in a QD, which can be formed due to the addition of an additional hole to a neutral acceptor. Exploring dependence of the impurity complex binding energy on such parameters of the matrix, surrounding the quantum dot, as temperature, the strength of the external electric field, the frequency of phonon modes, and the coupling constant with the contact medium (or with the heat-bath), is very important. It is also important to take into account the temperature dependence of the photoluminescence of the quantum dots under consideration [13,14,15,16,17]. As is known [18,19], for most semiconductor materials, the most significant contribution to the temperature dynamics of these QD’s energy levels is made by the electron—phonon interaction ([18,19]). A theoretical consideration of the temperature effect on the electronic energy levels in a semiconductor QD can be carried out by a statistical method (see, for example, [18]), under assumption that the main contribution to the temperature dependence is made by the electron-phonon interaction. The probability that an electron is in a state with energy *E_n_*, is given by the Fermi function

(1) $$f\left(\Psi \right)=\frac{1}{1+\exp\left[\frac{E_{n}-E_{F}}{kT}\right]}$$

where *E_F_*—is the Fermi energy, and the quantity *E_n_*, as is known, in quantum statistics has the meaning of free energy and is determined by the quantum canonical Gibbs distribution

(2) $$w\left(E\right)=\exp⟮\frac{E_{n}-E}{kT}⟯,$$

where *w*(*E*)—is the probability of a given discrete energy value *E*.

In our case, this is the electron energy *E_n_*, averaged over the vibrational states of the crystal lattice, defined as

(3) $$\exp⟮-\frac{E_{n}}{kT}⟯=\sum_{i=0}^{\infty }\exp⟮-\frac{E_{ni}}{kT}⟯,$$

here *E_ni_*—is the energy of an electron when it is in the *n*-th state, and the crystal lattice is in the *i*-th state. This energy is the sum of the electronic term *E_en_*, the phonon energy *E_p_*, and the energy of the electron-phonon interaction *E_ep_*:

(4) $$E_{ni}=E_{en}+E_{p}+E_{ep}.$$

If **q**—is the phonon wave vector, $\omega_{LA}\left(q\right),\omega_{TA}\left(q\right)$—frequencies of the longitudinal (*LA*) and transverse (*TA*) acoustic phonons, and $\hbar \omega_{LA}\left(q\right),\hbar \omega_{TA}\left(q\right)$—energies of the two-particle electron-phonon interaction, then for *E_p_* and *E_ep_* we can write

(5) $$E_{p}+E_{ep}=\int_{-\pi /a}^{\pi /a}\hbar \left[\omega_{LA}\left(q\right)+2\omega_{TA}\left(q\right)+\omega_{eLA}\left(q\right)+2\omega_{eTA}\left(q\right)\right]\left[N_{i}+\frac{1}{2}\right]dq,$$

here *a*—is the lattice constant, $N_{i}=0,1,2,\ldots$—phonon occupation numbers.

Integration in (5) requires knowledge of the corresponding dispersion laws. For three branches of acoustic phonons (longitudinal and two transversal), in the long-wave approximation, the linear dispersion law is valid

(6) $$\omega_{p}\left(q\right)=v_{j}q,$$

where $v_{j}$—is the sound velocity for the *j*-th phonon branch. 

The energy of the electron-phonon interaction is determined by the expression [20]

(7) $$\hbar \omega_{ep}\left(q\right)=\frac{\Xi }{v_{j}}\sqrt{\frac{kT}{2\rho V}}G,$$

where Ξ—is the deformation potential, ρ—the density of the QD material, G—the overlap integral.

Then, performing integration in (5), we obtain

(8) $$\exp⟮-\frac{E_{n}}{kT}⟯=\exp⟮-\frac{E_{en}}{kT}⟯\sum_{i=0}^{\infty }\exp⟮-\frac{\Omega }{\sqrt{kT}}⟮\frac{1}{v_{LA}}+\frac{2}{v_{TA}}⟯⟮N_{i}+\frac{1}{2}⟯⟯,$$

here, $v_{LA}$ and $v_{TA}$ are both the velocities of the longitudinal and transversal phonons and the next notation has been introduced

(9) $$\Omega =\frac{\pi \hbar \Xi G}{a}\sqrt{\frac{2}{\rho V}}.$$

Summing up in (8) for the temperature dependence of the *n*-th energy level, we obtain the expression

(10) $$E_{n}=E_{en}+kT\ln\left[4sh⟮\frac{\Omega }{v_{LA}\sqrt{kT}}⟯sh⟮\frac{2\Omega }{v_{TA}\sqrt{kT}}⟯\right].$$

As it has been mentioned above, it is important to take into account the temperature dependence of the photoluminescence of the quantum dots under consideration [13,14,15,16,17,20,21]. Let us further consider problem of the hole quasistationary states in an impurity complex $A^{+}+e$ in the semiconductor spherically symmetric QD. The interaction of an electron in the ground state in a QD with a hole localized at the $A^{0}$-center will be considered in framework of the adiabatic approximation [22]. In this case, the electron potential $V_{n,l,m}\left(\vec{r}\right)$, acting on the hole can be considered as averaged over the electron motion [22]

(11) $$V_{n,l,m}\left(\vec{r}\right)=-\frac{e^{2}}{4\pi \varepsilon_{0}\varepsilon }\int_{0}^{R_{0}}\frac{{\left|\Psi_{n,l,m}\left(\vec{r}\right)\right|}^{2}}{\left|\vec{r}-{\vec{r}}_{e}\right|}d{\vec{r}}_{e},$$

where *e* is the electron charge; *ε* is the dielectric constant of the QD material; *ε*_0_ is the electrical constant; $\Psi_{n,l,m}\left(\vec{r}\right)$ is the wave function of the QD electron; *m* = 0, ±1, ±2, … is the magnetic quantum number; *l* = 0, 1, 2, … is the orbital quantum number.

In the first order of the perturbation theory for the electron ground state (*m* = 0, *l* = 0) potential (11) can be written in the form

(12) $$V_{n,0,0}\left(r\right)=-\frac{e^{2}\beta_{n}}{4\pi \varepsilon_{0}\varepsilon R_{0}}+\frac{m_{h}^{*}\omega_{n}^{2}r^{2}}{2},$$

where $\beta_{n}=\gamma_{0}-Ci\left(2\pi n\right)+\ln\left(2\pi n\right)$; $\hbar \omega_{n}=\sqrt{2\hbar^{2}\pi^{2}n^{2}e^{2}/3m_{h}^{*}R_{0}^{3}4\pi \varepsilon_{0}\varepsilon }$; $\gamma_{0}=1.781$ is the Euler’s constant; Ci(x) denotes the integral cosine; *n* is the electron radial quantum number; $m_{h}^{*}$ is the hole effective mass. 

Since the confining potential of a QD, generally speaking, should have a finite depth, then in our model of the hole confinement potential (12), the potential amplitude *U*_0_ is an empirical parameter and satisfies the relation $U_{0}=-e^{2}\beta_{n}/4\pi \varepsilon_{0}\varepsilon R_{0}+m_{h}^{*}\omega_{n}^{2}R_{0}^{2}/2=m_{h}^{*}\omega_{0}^{2}R_{0}^{2}/2$, whence $\omega_{0}=\sqrt{\omega_{n}^{2}-e^{2}\beta_{n}/2\pi \varepsilon_{0}\varepsilon m_{h}R_{0}^{3}}$ is the characteristic frequency of the hole confining potential of a QD within the adiabatic approximation, in this case $U_{0}/\left(\hbar \;\omega_{\;0}\right)>>1$.

Usage of the adiabatic approximation makes it possible to take into account the effect of an external electric field on the hole bound states. Let the electric field strength ${\vec{E}}_{0}$ be directed along the *x* coordinate axis, then the oscillator energy levels (12), taking into account (10), are given in the form

(13) $$E_{n_{1},n_{2},n_{3}}^{n,\;0,\;0}\left(T\right)=-\frac{e^{2}}{\varepsilon R_{0}}\beta_{h}-\frac{{\left|e\right|}^{\;2}\;E_{0}^{2}}{2\;m_{h}\;\omega_{\;n}^{\;2}}+\hbar \omega_{n}⟮n_{1}+n_{2}+n_{3}+\frac{3}{2}⟯+kT\ln\left[4sh⟮\frac{\Omega }{v_{LA}\sqrt{kT}}⟯sh⟮\frac{2\Omega }{v_{TA}\sqrt{kT}}⟯\right]$$

and corresponding the one-particle wave functions are written as

(14) $$\Psi_{n_{1},\;n_{2},\;n_{3}}^{n}\left(x,y,z\right)=C_{n}\exp⟮-\frac{{\left(x-x_{0}\right)}^{2}+y^{2}+z^{2}}{2a_{n}^{2}}⟯H_{n_{1}}⟮\frac{x-x_{0}}{a_{n}}⟯H_{n_{2}}⟮\frac{y}{a_{n}}⟯H_{n_{3}}⟮\frac{z}{a_{n}}⟯,$$

where $C_{n}={\left[2^{n1+n2+n3}n_{1}!n_{2}!n_{3}!\pi^{3/2}a_{n}^{3}\right]}^{-1/2}$; $a_{n}=\sqrt{\hbar /\left(m_{h}^{*}\omega_{n}\right)}$; $x_{0}=\left|e\right|\;E_{0}/\left(m_{h}^{*}\;\omega_{n}^{2}\right)$; $H_{n}\left(x\right)$—Hermite polynomials; $n_{1}$,$n_{2}$,$n_{3}$—are the quantum numbers corresponding to the energy levels of the harmonic oscillator.

We will assume that the decay process of the quasi-stationary level of the $A^{+}$-center is due to dissipative tunneling.

The quantum tunneling with dissipation theory was actively developed in the early 1980-th in the works of A. I. Larkin et al. [1]. Interest in this theory was caused by the fact that for the case of a model potential of the “cubic parabola” type, describing Josephson contacts, for the case of a finite temperature, taking into account the heat-bath influence in the semiclassical instanton approximation, it was possible to obtain analytical results for the tunneling probability. The case of sufficiently low temperatures was considered in the works of A. J. Leggett, and at arbitrary temperatures and “viscosity coefficients” in the works of A. I. Larkin, Yu. N. Ovchinnikov and other authors [1]. In this case, the effects of dissipative tunneling were manifested in sufficiently small contacts, when, under certain conditions, voltage surges appeared [1]. An analogue of the Josephson effect can manifest itself in nuclear transformations, in which case the fission of atomic nuclei is interpreted as collective quantum tunneling with dissipation. In this case, role of the collective coordinate q is played by the quadrupole moment of the nucleus or its shape, and role of the heat-bath is played by single-nucleon excitations. Somewhat later, using the formalism developed in the works of A. I. Larkin, Yu. N. Ovchinnikov, B. I. Ivlev, A. J. Leggett, theory of quantum tunneling with dissipation for the case of 1D and 2D oscillator potentials was developed by A. A. Ovchinnikov, Yu. I. Dakhnovsky, V. A. Bendersky, E. I. Katz, et al. first to describe the kinetics of low-temperature adiabatic chemical reactions as tunnel systems with dissipation. Subsequently, the instanton method was generalized to the case of tunnel-coupled nanostructures. In particular, by combining the instanton method with the zero-range potential method, it was possible to obtain important analytical results describing the optical and transport properties of quantum molecules with impurity centers [1].

At the same time, a significant conclusion of the research was the statement that the above-mentioned optical and transport properties are significantly influenced not only by the dimension, size, shape of nanostructures, the presence of impurities, external fields, and final temperature but also by the thermostat matrix (or by the heat-bath) itself, in which these nanostructures are synthesized. In [1], when considering the experimentally observed 1D and 2D effects of dissipative tunneling within the limits of weak and strong dissipation, the field and temperature dependences of these effects were studied theoretically and experimentally, demonstrating their controllability. 

Thus, for metallic (golden and zirconium) quantum dots synthesized in a dielectric matrix, in a system of a combined atomic force and scanning tunneling microscope at a certain value of the external electric field strength, when the model double-well 1D-oscillator potential becomes symmetric, in the limit of weak dissipation in the field dependence of the tunneling probability, a single temperature-dependent peak is observed [1]. In the case of planar structures with synthesized QDs from colloidal gold, the field dependence of 2D dissipative tunneling can exhibit single and double bifurcations in the form of characteristic kinks, in the vicinity of which quantum beat regimes are realized [3]. In the strong dissipation limit for single InAs QDs, taking into account the influence of two local phonon modes on the field dependence of the probability of 1D dissipative tunneling, an oscillating mode with a nonequidistant spectrum of peaks was observed, which qualitatively coincides with the experimental I–V characteristic [2]. For structures with tunneling photodiodes based on tunneling coupled asymmetric InAs/GaAs quantum molecules, the experimental dependence of the photoconductivity qualitatively corresponded to the field dependence of the probability of 1D dissipative tunneling taking into account the influence of two local phonon modes (longitudinal and transversal optical phonons) [4].

It should be noted that in the one-instanton approximation, the decay probability (of dissipative tunneling), under conditions of an external electric field, can be represented in the form $\Gamma_{0}=B\exp\left(-S\right)$ (see also Appendix A), where S and B are determined as (in Bohr units) [1]:

(15) S=12⟮b′0+x0∗a′0+x0∗+1⟯ ⟮3−b′0+x0∗a′0+x0∗⟯ τ−0∗12 β∗⟮b′0+x0∗a′0+x0∗+1⟯2τ20∗−12γ∗⟮b′0+x0∗a′0+x0∗+1⟯2×⟮(1−x2∗)x1∗[cth(β∗x1∗)−1sh(β∗x1∗)(ch[(β∗−τ0∗)x1∗]−ch[β∗x1∗])+ch((β∗−τ0∗)x1∗)]−(1−x1∗)x2∗[cth(β∗x2∗)−1sh(β∗x2∗)(ch((β∗−τ0∗)x2∗)−ch(β∗x2∗))+ch((β∗−τ0∗)x2∗)]⟯

(16) $$\begin{array}{ll} B= & \frac{2E_{d}\sqrt{U_{0}^{*}}}{\hbar \;\sqrt{\pi }}\left(\frac{{b^{\prime }}_{0}+x_{0}^{*}}{{a^{\prime }}_{0}+x_{0}^{*}}+1\right)\sqrt{\varepsilon_{T}^{*}}\times \\ & \left\{A^{*}\left[\beta_{1}^{*}ch\left(\frac{\beta_{1}^{*}}{2}\right)-1\right]+D^{*}\left[\beta_{2}^{*}ch\left(\frac{\beta_{2}^{*}}{2}\right)-1\right]+A^{*}\left(1-\frac{\beta_{1}^{*}}{2}\frac{ch\left(\frac{\beta_{1}^{*}}{2}-\tau_{01}{}^{*}\right)}{sh\left(\frac{\beta_{1}^{*}}{2}\right)}\right)+D^{*}\left(\frac{\beta_{2}^{*}}{2}\frac{ch\left(\frac{\beta_{2}^{*}}{2}-\tau_{02}{}^{*}\right)}{sh\left(\frac{\beta_{2}^{*}}{2}\right)}-1\right)\right\}\times \\ & {\left(A^{*}\left[\frac{\beta_{1}^{*}}{2}\frac{ch\left(\frac{\beta_{1}^{*}}{2}-\tau_{01}{}^{*}\right)}{sh\left(\frac{\beta_{1}^{*}}{2}\right)}-1\right]+D^{*}\left[\frac{\beta_{2}^{*}}{2}\frac{ch\left(\frac{\beta_{2}^{*}}{2}-\tau_{02}{}^{*}\right)}{sh\left(\frac{\beta_{2}^{*}}{2}\right)}-1\right]\right)}^{-\frac{1}{2}} \end{array}$$

where

$x_{1,2}^{*}=\frac{1}{2}\left[\frac{\varepsilon_{L}^{*2}a^{*2}}{4U_{0}^{*}}+1+\frac{\varepsilon_{c}^{4}a^{*2}}{4\varepsilon_{L}^{*2}U_{0}^{*}}\mp \sqrt{{\left(\frac{\varepsilon_{L}^{*2}a^{*2}}{4U_{0}^{*}}+1+\frac{\varepsilon_{c}^{4}a^{*2}}{4\varepsilon_{L}^{*2}U_{0}^{*}}\right)}^{2}-\frac{\varepsilon_{L}^{*2}a^{*2}}{U_{0}^{*}}}\right]$,
$\gamma^{*}=\sqrt{{\left(\varepsilon_{L}^{*2}a^{*2}/\left(4\;U_{0}^{*}\right)+1+\varepsilon_{c}^{*\;4}a^{*2}/\left(4\;\varepsilon_{L}^{*2}U_{0}^{*}\right)\right)}^{2}-\varepsilon_{L}^{*2}a^{*2}/U_{0}^{*}}$,
$\tau_{0}^{*}=arcsh\left[⟮1-\frac{{b^{\prime }}_{0}+x_{0}^{*}}{{a^{\prime }}_{0}+x_{0}^{*}}⟯sh\left(\beta^{*}\right)/⟮1+\frac{{b^{\prime }}_{0}+x_{0}^{*}}{{a^{\prime }}_{0}+x_{0}^{*}}⟯\right]+\beta_{T}^{*}$,
$\varepsilon_{T}^{*}=kT/E_{h},\;\;\varepsilon_{L}^{*}=\hbar \omega_{L}/E_{h}$, $\varepsilon_{c}^{*}=\hbar \sqrt{c}/E_{d}$, $\beta_{T}^{*}=\sqrt{U_{0}^{*}}/a^{*}\varepsilon_{T}^{*}$, ${b^{\prime }}_{0}=b_{0}/a_{h}$, ${a^{\prime }}_{0}=a_{0}/a_{h}$,
$x_{\;0}{}^{*}=x_{0}/a_{h}$; $E_{h}\;\mathrm{and}\;a_{h}$ are the Bohr energy and hole radius, respectively;
$A^{*}=\left(2\;\varepsilon_{L}^{*2}a^{*2}-x_{1}^{*}\right)/\left(\left(x_{1}^{*}-x_{2}^{*}\right)x_{1}^{*}\right)$, $D^{*}=\left(2\;\varepsilon_{L}^{*2}a^{*2}-x_{2}^{*}\right)/\left(\left(x_{1}^{*}-x_{2}^{*}\right)x_{2}^{*}\right)$, $\beta_{1}^{*}=\sqrt{2\;U_{0}^{*}x_{1}^{*}}/\left(a^{*}\varepsilon_{T}^{*}\right)$,
$\beta_{2}^{*}=\sqrt{2\;U_{0}^{*}x_{2}^{*}}/\left(a^{*}\varepsilon_{T}^{*}\right)$, $\tau_{01}^{*}=\tau_{0}^{*}\sqrt{x_{1}^{*}}/\sqrt{2}$, $\tau_{02}^{*}=\tau_{0}^{*}\sqrt{x_{2}^{*}}/\sqrt{2}$.

Using the procedure of the zero-range potential method (see, for example, [23]), we obtain an equation that determines the hole energy dependence in the complex $A^{+}+e$ on temperature *T*, QD parameters, and on dissipative tunneling. The short-range potential of an impurity is modeled by the zero—range potential with intensity $\gamma =2\pi \hbar^{2}/(\alpha m_{h}^{*})$, which has the form:

(17) $$V_{\delta }\left(x,y,z;x_{a},y_{a},z_{a}\right)=\gamma \delta \left(x-x_{a}\right)\delta \left(y-y_{a}\right)\delta \left(z-z_{a}\right)\left[1+\left(r-r_{a}\right)\frac{\partial }{\partial r}\right],$$

where α is determined by the binding energy $E_{i}$ of the same $A^{+}$-center in a bulk semiconductor.

In the effective mass approximation, the wave function $\Psi_{\lambda h}\left(x,y,z;x_{a},y_{a},z_{a}\right)$ of an electron localized at a short-range potential satisfies the Schrödinger equation

(18) $$\left(E_{\lambda h}-H\right)\;\Psi_{\lambda h}\left(x,y,z;x_{a},y_{a},z_{a}\right)=V_{\delta }\left(x,y,z;x_{a},y_{a},z_{a}\right)\Psi_{\lambda h}\left(x,y,z;x_{a},y_{a},z_{a}\right),$$

where $E_{{}_{h}}^{QD}=-\hbar^{2}\lambda^{2}/\left(2m_{h}^{*}\right)$—the eigenvalues of the Hamilton operator $H^{\delta }=H+V_{\delta }\left(x,y,z;x_{a},y_{a},z_{a}\right)$; $H=-\;\hbar^{2}/\left(2\;m_{h}^{*}\right)\;\nabla^{\;2}+m_{h}^{*}\;\omega_{0}^{2}\;\left(x^{\;2}+y^{\;2}+z^{\;2}\right)/2-\left|e\right|\;E_{0}\;x$.

To determine the binding energy of a hole in a complex $A^{+}+e$ in the adiabatic approximation, it is necessary to construct a one-particle Green’s function $G\left(x,y,z;x_{a},y_{a},z_{a};E_{\lambda n}\right)$ to the Schrödinger equation with a Hamiltonian, containing potential (17)

(19) $$G\left(x,y,z;x_{a},y_{a},z_{a};E_{\lambda h}\right)=-\sum_{n1,n2,n3}\frac{\Psi_{n_{1},\;n_{2},\;n_{3}}^{n*}\left(x_{a},y_{a},z_{a}\right)\Psi_{n_{1},\;n_{2},\;n_{3}}^{n}\left(x,y,z\right)}{-E_{{}_{h}}^{QD}+i\hbar \Gamma_{0}+E_{n_{1},\;n_{2},\;n_{3}}^{n,\;0,\;0}\left(T\right)},$$

The Lippmann-Schwinger equation for a $A^{+}$-state in a QD with a parabolic confinement potential can be written as

(20) $$\Psi_{h}\left(x,y,z;x_{a},y_{a},z_{a}\right)=\int_{-\infty }^{+\infty }\int_{-\infty }^{+\infty }\int_{-\infty }^{+\infty }dx_{1}dy_{1}dz_{1}G\left(x,y,z;x_{a},y_{a},z_{a};E_{\lambda h}\right)\times V_{\delta }\left(x,y,z;x_{a},y_{a},z_{a}\right)\Psi_{\lambda h}\left(x_{1},y_{1},z_{1},x_{a},y_{a},z_{a}\right)$$

Substituting (17) into (20), we obtain

(21) $$\Psi_{h}\left(x,y,z;x_{a},y_{a},z_{a}\right)=\gamma G\left(x,y,z;x_{a},y_{a},z_{a};E_{\lambda h}\right)\left(T\Psi_{h}\right)\left(x,y,z;x_{a},y_{a},z_{a}\right),$$

where

(22) $$\left(T\Psi_{\lambda h}\right)\left(x,y,z;x_{a},y_{a},z_{a}\right)\equiv \underset{\begin{array}{l} r\to r_{a}\\ \varphi \to \varphi_{a}\\ \theta \to \theta_{a} \end{array}}{\lim}\left[1+(r-r_{a})\frac{\partial }{\partial r}\right]\Psi_{\lambda h}\left(x,y,z;x_{a},y_{a},z_{a}\right)$$

Acting as an operator T on both sides of relation (21), we obtain an equation that determines dependence of the bound state energy $E_{\lambda h}$ of the $A^{+}$-center on the QD parameters, on the position ${\vec{R}}_{\;a}=\left(x_{a},y_{a},z_{a}\right)$ of the impurity, and on the temperature *T*:

(23) $$\alpha =\frac{2\pi \hbar^{2}}{m_{h}^{*}}\left(TG\right)\left(x_{a},y_{a},z_{a};x_{a},y_{a},z_{a};E_{\lambda h}\right),$$

here α is determined by the energy $E_{\;i}$ of the bound state of the same $A^{+}$—the center in a massive semiconductor.

Then, for the Green’s function (19), taking into account (13) and (14), we obtain in the Bohr units

(24) $$\begin{array}{ll} G\left(x,y,z,x_{a},y_{a},z_{a};\eta_{\lambda h}^{2}\right)= & -\frac{1}{\pi^{\frac{3}{2}}a_{n}^{2}E_{h}}\exp⟮-\frac{{\left(x^{*}-x_{0}^{*}\right)}^{2}+y^{*2}+z^{*2}+{\left(x_{a}^{*}-x_{0}^{*}\right)}^{2}+y_{a}^{*2}+z_{a}^{*2}}{2}⟯\;\times \\ & \sum_{n_{1,}n_{2,}n_{3}}\frac{H_{n_{1}}\left(x^{*}-x_{0}^{*}\right)H_{n_{1}}\left(x_{a}^{*}-x_{0}^{*}\right)}{2^{n_{1}}!n_{1}!}\frac{H_{n_{2}}\left(y^{*}\right)H_{n_{2}}\left(y_{a}^{*}\right)}{2^{n_{2}}!n_{2}!}\frac{H_{n_{3}}\left(z^{*}\right)H_{n_{3}}\left(z_{a}^{*}\right)}{2^{n_{3}}!n_{3}!}\;\cdot \\ & {\left\{-\eta_{h}^{2}-\beta_{h}^{*}-\frac{x_{0}^{*2}}{4\beta^{-2}}+i4\Gamma_{0}^{*}+\beta^{-1}⟮n_{1}+n_{2}+n_{3}+\frac{3}{2}⟯+\frac{kT}{E_{h}}\ln\left[4sh⟮\frac{\Omega }{v_{LA}\sqrt{kT}}⟯sh⟮\frac{2\Omega }{v_{TA}\sqrt{kT}}⟯\right]\right\}}^{-1}, \end{array}$$

where the next notations are introduced $\eta_{h}^{2}=E_{{}_{h}}^{QD}/E_{h}$; $R_{0}^{*}=R_{0}/a_{h}$; $\beta =E_{h}/\hbar \omega_{n}$; $a_{n}^{*}=a_{n}/a_{h}$; $\beta_{h}^{*}=e^{2}\beta_{h}/\varepsilon R_{0}^{*}E_{h}a_{h}$; $\Gamma_{0}^{*}=\hbar \Gamma_{0}/4E_{h}$.

Further, given that

(25) $$\begin{array}{l} {\left(-\eta_{h}^{2}-\beta_{h}^{*}-\frac{x_{0}^{*2}\beta^{-2}}{4a_{h}^{*2}}+i4\Gamma_{0}^{*}+\frac{kT}{E_{h}}\ln\left[4sh\left(\frac{\Omega }{v_{LA}\sqrt{kT}}\right)sh\left(\frac{2\Omega }{v_{TA}\sqrt{kT}}\right)\right]+n_{1}+n_{2}+n_{3}+\frac{3}{2}\right)}^{-1}=\\ \beta^{-1}\int_{0}^{\infty }dt\exp\left[-t(-\beta \left(\eta_{h}^{2}+\beta_{h}^{*}+\frac{x_{0}^{*2}\beta^{-2}}{4a_{h}^{*2}}-i4\Gamma_{0}^{*}\right)+n_{1}+n_{2}+n_{3}+\frac{3}{2}+\frac{kT\beta }{E_{h}}\ln\left[4sh\left(\frac{\Omega }{v_{LA}\sqrt{kT}}\right)sh\left(\frac{2\Omega }{v_{TA}\sqrt{kT}}\right)\right]\right] \end{array}$$

expression for the Green’s function takes the following form

(26) $$\begin{array}{l} G\left(x,y,z,x_{a},y_{a},z_{a},\eta_{h}^{2}\right)=-\frac{1}{\pi^{\frac{3}{2}}a_{n}^{2}\beta E_{h}}\exp⟮-\frac{{\left(x^{*}-x_{0}^{*}\right)}^{2}+{\left(x_{a}^{*}-x_{0}^{*}\right)}^{2}+y^{2}+y_{a}^{2}+z^{2}+z_{a}^{2}}{2a_{n}}⟯\times \\ \int_{0}^{\infty }dt\exp[-t⟮-\beta ⟮\eta_{h}^{2}+\beta_{h}^{*}+\frac{x_{0}^{*2}\beta^{-2}}{4a_{h}^{*2}}-i4\Gamma_{0}^{*}⟯+\frac{kT\beta }{E_{h}}\ln\left[4sh⟮\frac{\Omega }{v_{LA}\sqrt{kT}}⟯sh⟮\frac{2\Omega }{v_{TA}\sqrt{kT}}⟯\right]+\frac{3}{2})⟯\times \;\\ \sum_{n_{1}=0}^{\infty }{⟮\frac{e^{-t}}{2}⟯}^{n_{1}}\frac{H_{n_{1}}⟮\frac{x-x_{0}}{a_{n}}⟯H_{n_{1}}⟮\frac{x_{a}-x_{0}}{a_{n}}⟯}{n_{1}!}\sum_{n_{2}=0}^{\infty }{⟮\frac{e^{-t}}{2}⟯}^{n_{2}}\frac{H_{n_{2}}⟮\frac{y}{a_{n}}⟯H_{n_{2}}⟮\frac{y_{a}}{a_{n}}⟯}{n_{2}!}\times \sum_{n_{3}=0}^{\infty }{⟮\frac{e^{-t}}{2}⟯}^{n_{3}}\frac{H_{n_{3}}⟮\frac{z}{a_{n}}⟯H_{n_{3}}⟮\frac{z_{a}}{a_{n}}⟯}{n_{3}!}. \end{array}$$

Taking summation over quantum numbers $n_{1},n_{2},n_{3}$ and separating the divergent part of the expression for the Green’s function (26), we obtain

(27) $$\begin{array}{ll} G\left(r,R_{a},\eta_{h}^{2}\right)= & -\frac{1}{\pi^{\frac{3}{2}}a_{n}^{2}\beta E_{h}}\int_{0}^{\infty }dt\exp[-t⟮-\beta ⟮\eta_{h}^{2}+\beta_{h}^{*}+\frac{x_{0}^{*2}\beta^{-2}}{4a_{h}^{*2}}-i4\Gamma_{0}^{*}⟯+\frac{3}{2}\;+\frac{kT\beta }{E_{h}}\ln\left[4sh⟮\frac{\Omega }{v_{LA}\sqrt{kT}}⟯sh⟮\frac{2\Omega }{v_{TA}\sqrt{kT}}⟯\right]⟯]\times \\ & \{{\left(1-\exp\left(-t\right)\right)}^{-\frac{3}{2}}\exp⟮-\frac{r^{2}+R_{a}^{2}}{2a_{n}}⟯\exp⟮\frac{2\left(rR_{a}\right)e^{-t}-\left(r^{2}+R_{a}^{2}\right)e^{-2t}}{a_{n}\left(1-e^{-2t}\right)}⟯-\frac{1}{t\sqrt{t}}\exp\left[-\frac{{\left(r-R_{a}\right)}^{2}}{2a_{n}^{2}t}\right]\}-\\ & \frac{\exp⟮-\sqrt{2⟮-\eta_{h}^{2}-\beta_{h}^{*}-\frac{x_{0}^{*2}\beta^{-2}}{4a_{h}^{*2}}+i4\Gamma_{0}^{*}+\frac{kT}{E_{h}}\ln\left[4sh⟮\frac{\Omega }{v_{LA}\sqrt{kT}}⟯sh⟮\frac{2\Omega }{v_{TA}\sqrt{kT}}⟯\right]+\frac{3}{2}⟯}\frac{\left|r-R_{a}\right|}{a_{n}}⟯}{2\pi^{\frac{3}{2}}a_{n}\beta E_{h}\left|r-R_{a}\right|} \end{array}$$

Substituting (27) into (23), we obtain the dispersion equation that determines dependence of the binding energy $E_{{}_{h}}^{QD}$ of a hole in the $A^{+}+e$ complex on the QD parameters, on temperature *T*, and on the electron quantum number *n*

(28) $$\begin{array}{ll} \eta_{i}= & \sqrt{-\eta_{h}^{2}-\beta_{h}^{*}-\frac{x_{0}^{*2}\beta^{-2}}{4a_{h}^{*2}}+i4\Gamma_{0}^{*}+\frac{kT}{E_{h}}\ln\left[4sh⟮\frac{\Omega }{v_{LA}\sqrt{kT}}⟯sh⟮\frac{2\Omega }{v_{TA}\sqrt{kT}}⟯\right]+\frac{3}{2}}+\\ & \sqrt{\frac{2}{\beta \pi }}\int_{0}^{\infty }dt\exp\{-t\beta ⟮-\eta_{h}^{2}-\beta_{h}^{*}-\frac{x_{0}^{*2}\beta^{-2}}{4a_{h}^{*2}}+i4\Gamma_{0}^{*}+\frac{3}{2\beta }+\frac{kT}{E_{h}}\times \\ & \ln\left[4sh⟮\frac{\Omega }{v_{LA}\sqrt{kT}}⟯sh⟮\frac{2\Omega }{v_{TA}\sqrt{kT}}⟯\right]⟯\}\left[\frac{1}{2t\sqrt{2t}}-{\left(1-e^{-2t}\right)}^{-\frac{3}{2}}\exp⟮-\frac{R_{a}^{{*}^{2}}\beta^{-1}}{2}\times \frac{1-e^{-t}}{1+e^{-t}}⟯\right] \end{array}$$

where $R_{0}^{*}=R_{0}/a_{h}$; $\eta_{i}=\sqrt{\left|E_{i}\right|/E_{h}}$.

**Figure 2:**
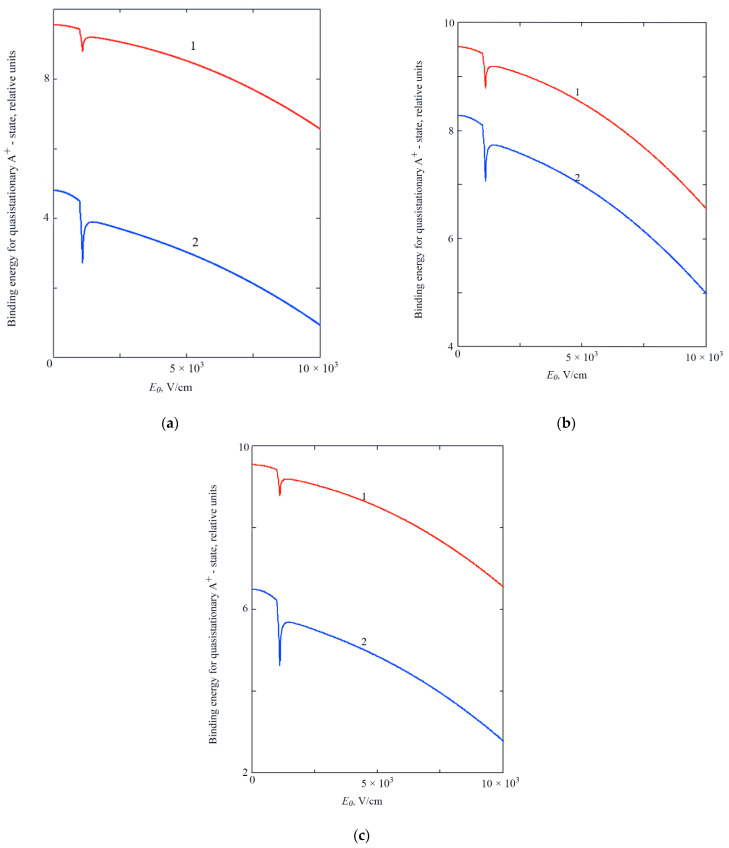
Dependence of the binding energy of the quasi-stationary state of a hole in a complex $A^{+}\;+\;e$ on the strength of the external electric field *E*_0_, at $R_{0}^{*}=1$; $U_{0}^{*}=300$: (**a**)—for different temperatures 1—T = 100 K; 2—T = 300 K; at $\varepsilon_{L}=0.5$; $\varepsilon_{C}=1\;$; (**b**)—for different values of the parameter $\varepsilon_{L}$ that determines the frequency of the phonon mode: 1—$\varepsilon_{L}=0.5$, 2—$\varepsilon_{L}=1$; at $\varepsilon_{C}=1\;$; T=100 K; (**c**)—for different values of the parameter $\varepsilon_{C}$ that determines the constant of interaction with the contact medium (with a thermostat or with the heat-bath): 1—$\varepsilon_{C}=1$, 2—$\varepsilon_{C}=0.5$; at $\varepsilon_{L}=0.5$; T = 100 K.

It should be noted that the hole binding energy in the considered case is a complex quantity. Its real part determines the average binding energy of the resonance state of the $A^{+}$-center, EhQD¯=ReEhQD, and its doubled imaginary part determines the broadening of the corresponding energy level ΔEh=2ImEhQD. Figure 2a–c shows the result of a numerical analysis of the dispersion Equation (28) for the case of a centered $A^{+}$-center ($R_{a}^{*}=0$) at different values of the QD radius $R_{0}^{*}$. It was taken into account that the binding energy of the $A^{+}$-state is measured from the ground state level of the adiabatic oscillatory well.

As it can be seen from Figure 2a–c, that in the field dependence of the binding energy of the *A^+^*-state, there are “dips” at a fixed temperature. This is due to the “tuning” effect of the starting energy level of the quasistationary *A^+^*-state to the states, caused by the electron—phonon interaction in the matrix, surrounding the quantum dot, i.e., with the effect of resonant tunneling. The dip depth increases with increasing temperature, which is due to dynamics of the temperature-dependent peak in the field dependence of the dissipative tunneling probability [1].

A decrease in the binding energy of the *A^+^*-state with an increase in the strength of the external electric field is associated with the Stark shift in energy and with the polarization of the *A^+^*-center, and with an increase in temperature—with broadening of energy levels and the corresponding “spreading” of the wave function of the *A^+^*-state. One can also see (see Figure 2b,c) a sufficiently high sensitivity of the binding energy of the *A^+^*-state to the frequency of the phonon mode (Figure 1b) and to the constant of interaction with the contact medium (Figure 2c). An increase in the latter constant leads to blocking of tunneling decay and to a corresponding increase in the binding energy of the *A^+^*-state (compare curves 1 and 2 in Figure 2c).

### 3.2. Dependence of the Spectral Intensity for Recombination Radiation in a Quantum Dot with an Impurity Complex on the Parameters of the Surrounding Matrix (of the Heat-Bath)

Let us consider the process of radiative transition of an excited electron to a level of $A^{+}$-center. The energy structure of the considered system “quantum dot with impurity complex *A^+^ + e*” under conditions of dissipative tunneling is shown in Figure 1. The Coulomb interaction of an electron with a hole is accompanied by a radiative transition of an electron to an energy level of $A^{+}$-center with the radiation wavelength λ. Estimates have shown that, depending on the QD parameters and on the adiabatic potential of the electron, as well as on the temperature and on strength of the external electric field, the wavelength of the recombination radiation can vary in the range λ = 0.1–70 μm.

The energy spectrum of an electron in the size-quantized band, taking into account (10), is written in the form

(29) $$E_{n,l}=\frac{{\tilde{X}}_{n,\;l}^{2}E_{h}}{R_{0}^{*2}}+kT\ln\left[4sh\left(\frac{\Omega }{v_{LA}\sqrt{kT}}\right)sh\left(\frac{2\Omega }{v_{TA}\sqrt{kT}}\right)\right],$$

here ${\tilde{X}}_{n,l}$ is the root of the Bessel function of half-integer order l+1/2.

The wave function of an electron is given by an expression of the next form

(30) $$\Psi_{n,l,m}\left(r,\theta ,\varphi \right)=Y_{l,m}\left(\theta ,\varphi \right)\frac{J_{l+\frac{3}{2}}\left(K_{n,l}r^{*}\right)}{a_{h}^{\frac{3}{2}}\sqrt{2\pi }R_{0}^{*}\sqrt{r^{*}}J_{l+\frac{3}{2}}\left(K_{n,l}R_{0}^{*}\right)},$$

where $K_{n,l}$ is determined by the following expression

$$K_{n,l}=\sqrt{\frac{{\tilde{X}}_{n,\;l}^{2}}{R_{0}^{*2}}+\frac{kT}{E_{h}}\ln\left[4sh⟮\frac{\Omega }{v_{LA}\sqrt{kT}}⟯sh⟮\frac{2\Omega }{v_{TA}\sqrt{kT}}⟯\right]}$$

Spectral Intensity of Recombination Radiation (SIRR), taking into account the dispersion of QD sizes and the finite lifetime of the resonant $A^{+}$-state, is determined by an expression of the next form [24]

(31) $$\Phi \left(\omega \right)=\frac{4\omega^{2}\sqrt{\varepsilon }e^{2}}{c^{3}V}\left|\frac{P_{eh}e_{0}}{m_{0}}\right|\int \sum_{nlm}{\left|M\right|}^{2}P\left(u\right)\frac{\Gamma_{0}}{\frac{\hbar^{2}\Gamma_{0}^{2}}{4}+{\left(E_{nlm}-E_{\lambda h}-\hbar \;\omega \right)}^{2}}du,$$

where $m_{0}$ is the mass of a free electron; $P_{eh}$ is the matrix element of the momentum operator at the Bloch amplitudes of band carriers; ω—the frequency of the emitted electromagnetic wave with polarization $e_{0}$; V—is the QD volume; P(u)—the Lifshits—Slyozov function [25]:

(32) $$P\left(u\right)=\{\begin{array}{l} \frac{3^{4}e\;u^{2}\exp\left[-1/\left(1-2\;u/3\right)\right]}{2^{\;\frac{5}{3}}{\left(u+3\right)}^{\frac{7}{3}}\;{\left(3/2-u\right)}^{\frac{11}{3}}},\;u<\frac{3}{2},\\ 0,\;\;\;\;\;\;\;u>\frac{3}{2}. \end{array}$$

The wave function of $A^{+}$-state, in the case of a central location of the $A^{+}$-center QD, has the next form (see (27)):

(33) $$\begin{array}{ll} \Psi_{h}\left(r\right)= & -C\int_{0}^{\infty }dt\exp\left\{-\beta t⟮-\eta_{h}^{2}-\beta_{h}^{*}-\frac{x_{0}^{*2}}{a_{n}^{*4}}+i4\Gamma_{0}^{*}+\frac{kT}{E_{h}\beta }\ln\left[4sh⟮\frac{\Omega }{v_{LA}\sqrt{kT}}⟯sh⟮\frac{2\Omega }{v_{TA}\sqrt{kT}}⟯\right]+\frac{3}{2\beta }⟯\right\}\times \\ & {\left(1-\exp\left(-t\right)\right)}^{-\frac{3}{2}}\exp⟮-\frac{\left(1+e^{-2t}\right)}{\left(1-e^{-2t}\right)}\times \frac{r^{2}}{2a_{n}^{2}}⟯ \end{array}$$

where C is the normalization factor determined by an expression of the next form

(34) $$C={\left\{\frac{2\sqrt{\pi }}{{\left[\eta_{h}^{2}\left(T\right)\right]}^{2}}\frac{\Gamma ⟮1+\frac{\eta_{h}^{2}\left(T\right)}{2}⟯\cdot a_{n}^{3}}{\Gamma ⟮\frac{\eta_{h}^{2}\left(T\right)-1}{2}⟯}\left[\frac{\eta_{h}^{2}\left(T\right)}{2}⟮\Psi ⟮\frac{\eta_{h}^{2}\left(T\right)}{2}+1⟯-\Psi ⟮\frac{\eta_{h}^{2}\left(T\right)}{2}-\frac{1}{2}⟯-1⟯\right]\right\}}^{-\frac{1}{2}},$$

here $\eta_{\lambda h}^{2}\left(T\right)$—is determined by the dispersion Equation (28).

The matrix element of the radiative transition of an excited electron to the $A^{+}$-center level is given by the next expression

(35) $$M=i\;\lambda_{0}\sqrt{\frac{2\pi \alpha^{*}I_{0}}{\omega }}\;\left(E_{\;n,l,m}-E_{h}\right)\langle \Psi_{h}\left(r\right)\left|\;\left({\vec{e}}_{\lambda },\vec{r}\right)\;\right|\Psi_{n,l,m}\left(\rho ,\theta ,\varphi \right)\rangle .$$

Taking into account (29), (30), and (33), the matrix element of the radiative recombination transition of an electron from the ground state of the size-quantized band to the $A^{+}$-center level in the QD can be represented in the next form

(36) $$\begin{array}{ll} M= & \frac{a_{h}^{-1}2^{\;-\frac{3}{2}}\pi^{\;-\frac{5}{4}}\sqrt{3}\;\beta^{\frac{1}{2}}}{\sqrt{2\pi }R_{0}^{*4}J_{\frac{3}{2}}\left({\tilde{X}}_{n,1}\right)}[\frac{\Gamma ⟮1+\frac{\eta_{h}^{2}\left(T\right)}{2}⟯}{{\left[\eta_{h}^{2}\left(T\right)\right]}^{2}\Gamma ⟮\frac{\eta_{h}^{2}\left(T\right)-1}{2}⟯}\times \\ & {\left[\frac{\eta_{h}^{2}\left(T\right)}{2}⟮\Psi ⟮\frac{\eta_{h}^{2}\left(T\right)}{2}+1⟯-\Psi ⟮\frac{\eta_{h}^{2}\left(T\right)}{2}-\frac{1}{2}⟯-1⟯\right]\;]}^{\;-\;\frac{1}{2}}\int_{0}^{+\infty }\int_{-\pi }^{+\pi }\int_{0}^{2\pi }r^{*2}dr^{*}\cos\theta \sin\theta d\theta d\varphi \times \\ & \int_{0}^{\infty }dt\exp\left[-t⟮-\eta_{h}^{2}\beta -\beta \beta_{h}^{*}-\frac{x_{0}^{*2}\beta }{a_{n}^{*4}}+i4\beta \Gamma_{0}^{*}+\frac{kT\beta }{E_{h}}\ln\left[4sh⟮\frac{\Omega_{n}}{v_{LA}\sqrt{kT}}⟯sh⟮\frac{2\Omega_{n}}{v_{TA}\sqrt{kT}}⟯\right]+\frac{3}{2}⟯\right]\times \;\\ & {\left(1-\exp\left(-t\right)\right)}^{-\frac{3}{2}}\exp⟮-\frac{\left(1+e^{-2t}\right)}{\left(1-e^{-2t}\right)}\times \frac{r^{*2}}{2}⟯Y_{l,m}\left(\theta ,\varphi \right)\frac{J_{l+\frac{3}{2}}⟮\frac{{\tilde{X}}_{n,l}}{R_{0}^{*}}r^{*}⟯}{\sqrt{r^{*}}J_{l+\frac{3}{2}}\left({\tilde{X}}_{n,l}\right)}, \end{array}$$

where $R_{0}^{*}=R_{0}/a_{h}$—the QD radius.

Calculation of (36) leads to integrals, giving the selection rules for quantum numbers *m* and *l*:

(37) $$\int_{0}^{2\pi }\exp\left(\mathrm{im}\varphi \right)d\varphi =\left\{ \begin{array}{l} 2\pi ,\;\mathrm{if}\;m=0\\ 0,\;\mathrm{if}\;m\neq 0, \end{array}\right.$$

(38) $$\int_{0}^{\pi }P_{l}\left(\cos\theta \right)\cos\theta \sin\theta d\theta =\left\{ \begin{array}{l} \frac{2}{3},\;\mathrm{if}\;l=1\\ 0,\;\mathrm{if}\;l\neq 1. \end{array}\right.$$

Thus, the radiative transition of an electron to the $A^{+}$-center level is possible only from states with the values of quantum numbers *l* = 1 and *m* = 0.

The remaining integral over the radial coordinate $r^{*}$ has the next form

(39) $$\int_{0}^{\infty }dr^{*}r^{*\frac{3}{2}}J_{l+\frac{3}{2}}⟮\frac{{\tilde{X}}_{n,l}r^{*}}{R_{0}^{*}}⟯\exp⟮-\frac{\left(1+e^{-2t}\right)}{2\left(1-e^{-2t}\right)}r^{*2}⟯=\sqrt{\frac{{\tilde{X}}_{n,l}}{R_{0}^{*}}}\exp⟮-\frac{1}{2}⟮\frac{1-e^{-2t}}{1+e^{-2t}}⟯{⟮\frac{{\tilde{X}}_{n,l}}{R_{0}^{*}}⟯}^{2})\times {⟮\frac{1-e^{-2t}}{1+e^{-2t}}⟯}^{\frac{3}{2}}.$$

Taking into account (37), (38), and (39), for the square of the matrix element (36) we have

(40) $$\begin{array}{ll} {\left|M\right|}^{2}= & \frac{\;\beta_{h}{\tilde{X}}_{n,1}}{2^{2}\pi^{\;\frac{5}{2}}a_{h}^{2}R_{0}^{*9}{\left|J_{\frac{3}{2}}\left({\tilde{X}}_{n,1}\right)J_{\frac{5}{2}}\left({\tilde{X}}_{n,1}\right)\right|}^{2}}|\frac{\Gamma ⟮1+\frac{\eta_{h}^{2}}{2}⟯}{{\left[\eta_{h}^{2}\right]}^{2}\Gamma ⟮\frac{\eta_{h}^{2}-1}{2}⟯}\times \\ & {\left(1-\exp\left(-t\right)\right)}^{-\frac{3}{2}}\exp⟮-\frac{\left(1+e^{-2t}\right)}{\left(1-e^{-2t}\right)}\times \frac{r^{*2}}{2}⟯Y_{l,m}\left(\theta ,\varphi \right)\frac{J_{l+\frac{3}{2}}⟮\frac{{\tilde{X}}_{n,l}}{R_{0}^{*}}r^{*}⟯}{\sqrt{r^{*}}J_{l+\frac{3}{2}}\left({\tilde{X}}_{n,l}\right)}\\ & \times |\int_{0}^{\infty }dt\exp\left[-t⟮-\eta_{h}^{2}\beta -\beta \beta_{h}^{*}-\frac{x_{0}^{*2}\beta }{a_{n}^{*4}}+i4\beta \Gamma_{0}^{*}+\frac{kT\beta }{E_{h}}\ln\left[4sh⟮\frac{\Omega }{v_{LA}\sqrt{kT}}⟯sh⟮\frac{2\Omega }{v_{TA}\sqrt{kT}}⟯\right]+\frac{3}{2}⟯\right]\times \\ & {{\left(1-\exp\left(-t\right)\right)}^{-\frac{3}{2}}\exp⟮-\frac{1}{2}⟮\frac{1-e^{-2t}}{1+e^{-2t}}⟯{⟮\frac{{\tilde{X}}_{n,l}}{R_{0}^{*}}⟯}^{2}⟯{⟮\frac{1-e^{-2t}}{1+e^{-2t}}⟯}^{\frac{3}{2}}|}^{2} \end{array}$$

Taking into account (32) and (40) for the spectral intensity of recombination radiation (SIRR) in QD (31), we can write

(41) $$\begin{array}{ll} \Phi \left(X,T\right)= & \Phi_{0}\times \frac{\;a_{h}^{4}\beta_{h}{\tilde{X}}_{n,1}X^{2}}{R_{0}^{*12}{\left|J_{\frac{3}{2}}\left({\tilde{X}}_{n,1}\right)J_{\frac{5}{2}}\left({\tilde{X}}_{n,1}\right)\right|}^{2}}\times \int_{0}^{\frac{3}{2}}duP\left(u\right)\times \\ & {\left|\frac{\Gamma ⟮1+\frac{\eta_{h}^{2}}{2}⟯}{{\left(\eta_{h}^{2}\right)}^{2}\Gamma ⟮\frac{\eta_{h}^{2}-1}{2}⟯}\left[\frac{\eta_{h}^{2}}{2}(\Psi ⟮\frac{\eta_{h}^{2}}{2}+1⟯-\Psi \left(\frac{\eta_{h}^{2}}{2}-\frac{1}{2}\right)-1⟯\right]\right|}^{-1}\times \\ & |\int_{0}^{\infty }dt\exp\left[-t⟮-\eta_{h}^{2}\beta -\beta \beta_{h}^{*}-\frac{x_{0}^{*2}\beta }{a_{n}^{*4}}+i4\beta \Gamma_{0}^{*}+\frac{kT\beta }{E_{h}}\ln\left[4sh⟮\frac{\Omega }{v_{LA}\sqrt{kT}}⟯sh⟮\frac{2\Omega }{v_{TA}\sqrt{kT}}⟯\right]+\frac{3}{2}⟯\right]\times \\ & {{\left(1-\exp\left(-t\right)\right)}^{-\frac{3}{2}}\exp⟮-\frac{1}{2}⟮\frac{1-e^{-2t}}{1+e^{-2t}}⟯{⟮\frac{{\tilde{X}}_{n,l}}{R_{0}^{*}}⟯}^{2}⟯{⟮\frac{1-e^{-2t}}{1+e^{-2t}}⟯}^{\frac{3}{2}}|}^{2}\times \\ & \frac{\Gamma_{0}^{*}}{\Gamma_{0}^{*2}+{⟮\frac{X_{n,1}^{2}}{R_{0}^{*2}}+\frac{kT}{E_{h}}\ln\left[4sh⟮\frac{\Omega }{v_{LA}\sqrt{kT}}⟯sh⟮\frac{2\Omega }{v_{TA}\sqrt{kT}}⟯\right]-\eta_{\lambda h}^{2}-X)}^{2}}, \end{array}$$

where $X=\hbar \omega /E_{h}$; $\Phi_{0}=\sqrt{\varepsilon }e^{2}\left|P_{eh}e_{0}\right|/4\pi^{\;\frac{5}{2}}\hbar^{3}c^{3}m_{0}$.

**Figure 3:**
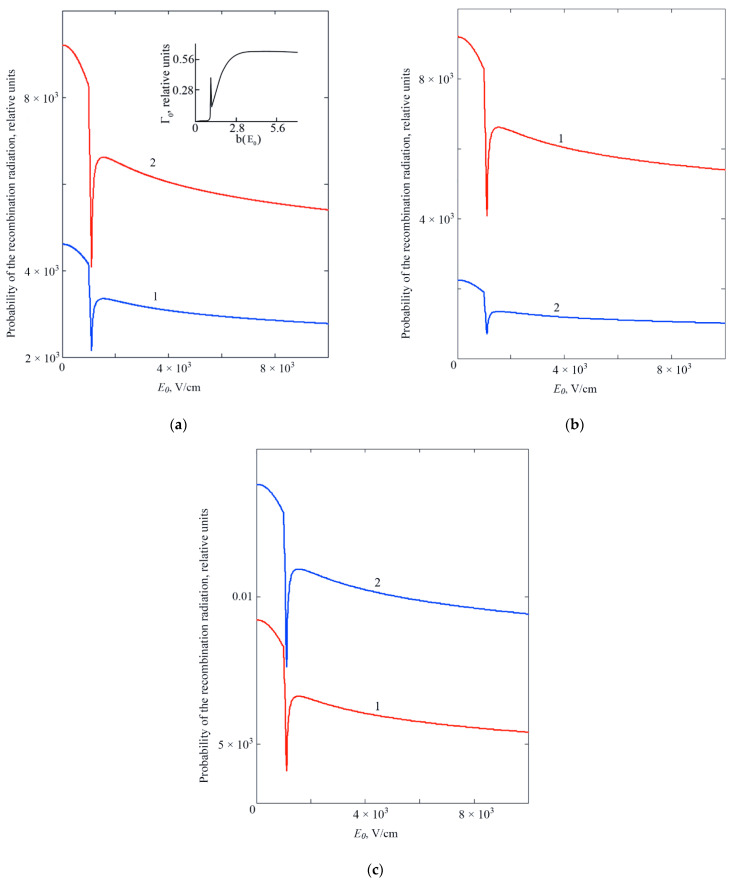
Dependence of the SIRR on the strength of the external electric field $E_{0}$ at ${\bar{R}}_{0}^{*}=1$;$U_{0}^{*}=300$; $\eta_{i}=4$: (**a**) for various values of temperature, *T,* K: 1—100, 2—300; $\varepsilon_{L}=0.5$; $\varepsilon_{C}=1$; (**b**)—for different values of the parameter $\varepsilon_{L}$ that determines frequency of the phonon mode: 1—0.5; 2—1; $\varepsilon_{C}=1$; T = 100 K; (**c**)—for different values of the parameter $\varepsilon_{C}$ that determines constant of interaction with the contact medium (with the heat-bath): 1—1.0; 2—0.5; $\varepsilon_{L}=1$, T = 100 K. The inset in Figure 3a shows dependence of the dissipative tunneling probability Γ_0_ on the parameter “b”, which determines the strength of the external electric field, obtained in [1].

Figure 3 a–c shows the SIRR dependence on the magnitude of the external electric field $E_{0}$. It can be seen that the decrease in the SIRR value with increasing of $E_{0}$ is accompanied by “dips”, that appear at certain values of the external electric field strength and temperature. In [1] it is shown that variation of the strength of the external electric field can lead to transformation of the shape of the double-well oscillatory potential, which simulates the system “QD—surrounding matrix”, while the transition to the symmetric shape of the double-well oscillatory potential is accompanied by the appearance of a peak in the field dependence of tunneling probability (see inset in Figure 3a). Thus, the nature of the dip appears to be related to the effect of resonant tunneling, when the double-well oscillator potential becomes symmetric.

An increase in the SIRR value with the temperature increasing (compare curves 1 and 2 in Figure 3a) is associated with an increase in the overlap integral of the wave functions of the initial and final states due to temperature smearing of energy levels. It should be noted that the presence of dissipative tunneling makes the optics of quantum dots very sensitive to the parameters of the surrounding matrix, which determine, respectively, the frequency of the phonon mode (see Figure 3b,c) *ɛ*_L_ and the constant of interaction with the contact medium (with the heat-bath) *ɛ_C_*. With an increase in the value of *ɛ_L_*, the wave function of the $A^{+}$-state “spreads” due to the electron—phonon interaction, which is accompanied by a decrease in the SIRR value. An increase in the parameter *ɛ_C_* leads to an increase in the “viscosity” of the surrounding matrix, i.e., to a decrease in the probability of dissipative tunneling. As a result, the binding energy of the $A^{+}$-state increases, and the overlap integral of the wave functions of the initial and final states decreases, which leads to a decrease in the SIRR value.

When constructing the figures, the task was to show the features of the field dependence of the binding energy of the quasi-stationary state of the hole in the complex $A^{+}+e$ and the spectral intensity of recombination radiation (SIRR) associated with the dynamics of the temperature-dependent peak of the dissipative tunneling probability (see, for example, [1]). The choice of such parameter values is related, firstly, to the fact that InSb was chosen as the QD material; therefore, the parameters were expressed in Bohr units: the effective Bohr radius $a_{d}\approx 65$ nm and the effective Bohr energy $E_{d}\approx 0.001$ eV. Secondly, the choice of the numerical values of the parameters in Bohr units was carried out by numerical studies of the possibility of identifying the indicated features of the curves.

The high sensitivity of the spectra of recombination radiation and dissipative tunneling to the strength of an external electric field inspires a certain optimism for using the considered system “*A^+^ + e*” (a hole localized on a neutral acceptor interacting with an electron localized in the ground state of a quantum dot) for the diagnosis of amino acids and other ligands conjugated or surrounding QDs.

## 4. Discussion and Conclusions

Within the framework of the zero-radius potential model in the adiabatic approximation in combination with the one-instanton method, an analytical solution of the problem for bound states of a hole localized at a neutral acceptor interacting with an electron localized in the ground state of a spherically symmetric quantum dot in the presence of dissipative tunneling in an external electric field has been obtained. The solution of this problem made it possible to calculate in the dipole approximation the field dependence of the intensity of recombination radiation associated with the optical transition of an electron from the ground state of a quantum dot to the quasi-stationary state of the *A^+^*- center. It is shown that the field dependence of both the binding energy of the *A^+^*- state and the recombination radiation contains “dips” that appear at certain values of the external electric field strength and temperature. The “dips” are associated with the transformation of the shape of the double-well oscillatory potential, which models the “quantum dot—surrounding matrix” system, while a significant increase in the probability of dissipative tunneling is interpreted as the effect of resonant tunneling. Studies of the curves revealed a rather high sensitivity of SIRR to such parameters of the surrounding matrix as temperature, the constant of interaction with the contact medium, the frequency of phonon modes, and the strength of the external electric field, which makes recombination radiation in the system under consideration attractive for obtaining information about the medium surrounding the quantum dot. Using the above SIRR calculations, one can obtain a formula for the dependence of the threshold value of the photon energy in recombination radiation on the magnitude of the external electric field.

The threshold value of the recombination radiation energy is defined as the sum

$${\left(\hbar \omega \right)}_{th}=E_{e}+E_{\lambda h}+E_{g}$$

where

$$E_{e}=\frac{{\tilde{X}}_{n,\;l}^{2}E_{h}}{R_{0}^{*2}}+kT\ln\left[4sh\left(\frac{\Omega }{v_{LA}\sqrt{kT}}\right)sh\left(\frac{2\Omega }{v_{TA}\sqrt{kT}}\right)\right]$$

− energy of the ground state level of an electron in a QD, taking into account its temperature dependence,$R_{0}^{*}=R_{0}/a_{h}$—the QD radius; ${\tilde{X}}_{n,\;1}$—root of the n-th order for Bessel function. $E_{\lambda h}$—the binding energy of the *A^+^*-center, determined by the dispersion Equation (28), $E_{g}$—band gap in a bulk semiconductor.

Then, in Bohr units, the threshold value of the recombination radiation energy can be written as

(42) $$X_{th}=\frac{{\tilde{X}}_{1,1}^{2}}{R_{0}^{*2}}+\frac{kT}{E_{h}}\ln\left[4sh\left(\frac{\Omega }{v_{LA}\sqrt{kT}}\right)sh\left(\frac{2\Omega }{v_{TA}\sqrt{kT}}\right)\right]+\frac{E_{g}}{E_{h}}+\eta_{\lambda h}^{2},,$$

where $X_{th}={\left(\hbar \omega \right)}_{th}/E_{h}$, Ω− parameter depending on the deformation potential of the QD material;$v_{LA},v_{TA}-$ velocities of longitudinal and transverse phonons; $\eta_{\lambda h}^{2}=E_{\lambda h}/E_{h}$; $a_{h},E_{h}-$ the Bohr radius and Bohr energy, respectively. For QD based on InSb at T=100 K; $U_{0}=0.3$ eV;$R_{0}=70$ nm; $\varepsilon_{L}=0.5$; $\varepsilon_{C}=1$, change in electric field strength in the range from 0 to $E_{0}=10^{4}$ V/cm leads to a shift in the threshold value of the energy of recombination radiation calculated by formula (42) and the SIRR maximum by approximately 30 meV.

Thus, in the presence of dissipative tunneling in an external electric field, the “quantum dot–impurity complex” system investigated in this work can be used to diagnose amino acids. Indeed, amino acids can be negatively or positively charged, they can interact with quantum dots, modifying their energy spectrum and impurity states and hence the spectrum of recombination radiation. It should be noted that earlier in [6], the question of the advantages of using semiconductor quantum dots for the study and diagnostics of biological systems has been discussed. In this case, the process of tunneling between the core-shell quantum dots and a biological object was not taken into account by the authors of [6].

As our studies have shown, the parameters of the surrounding matrix can have a significant effect on the value of SIRR due to the change in the probability of dissipative tunneling. This is important because it becomes possible to obtain additional information about biological objects.

## Figures and Tables

**Figure A1:**
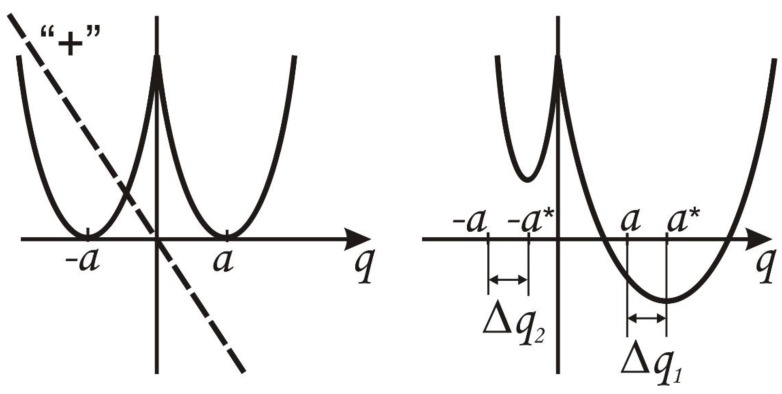
Effect of an electric field on a symmetric double-well oscillatory potential.

**Figure A2:**
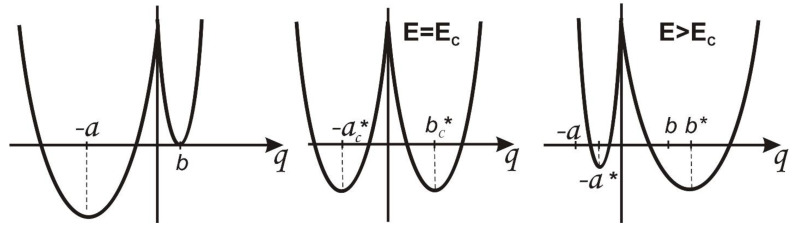
Effect of an electric field on an asymmetric double-well oscillatory potential.

**Figure A3:**
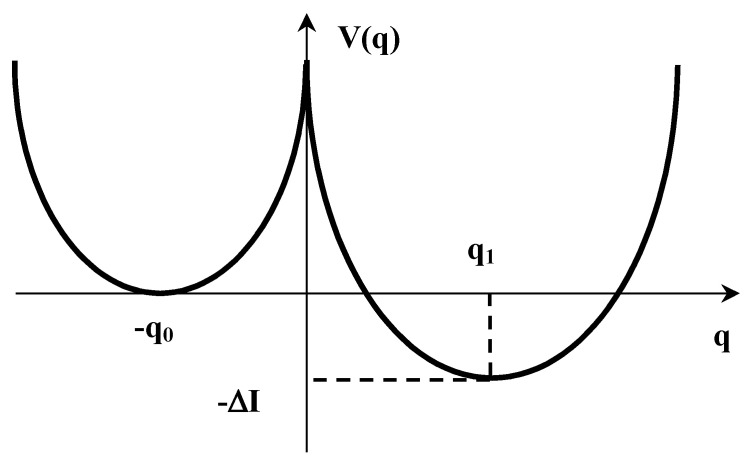
Potential energy of a particle along the tunneling coordinate.

**Figure A4:**
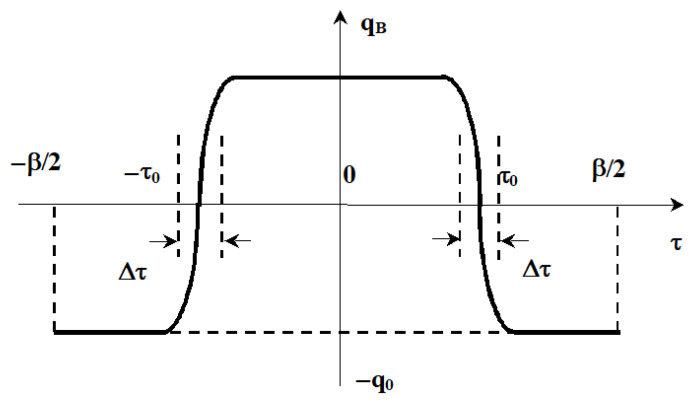
Instanton trajectory $q_{B}(\tau )$.

## Appendix A

**1D—dissipative tunneling in an external electric field. The role of the thermostat (of the heat-bath)**, (see also [1,26]).

Let us consider the influence of an electric field on a two-well model oscillatory 1D potential (Figure A1).

Taking into account the influence of an electric field on the symmetric double-well model oscillatory potential can be represented in the next form:

(A1) $$U(q)=\frac{\omega_{0}^{2}}{2}{\left(q-a\right)}^{2}\theta (q)+\frac{\omega_{0}^{2}}{2}{\left(q+a\right)}^{2}\theta (-q)-|e|Eq.$$

The electric field changes the symmetry of the potential and the minima shift:

$$(1)\;q>0;\;U_{1}=\frac{\omega_{0}^{2}}{2}{\left(q-a\right)}^{2}-\left|e\right|Eq=\frac{\omega_{0}^{2}}{2}{\left(q-a*\right)}^{2}-a\left|e\right|Eq-\frac{\left|e\right|E}{2\omega_{0}^{2}},\;\mathrm{where}\;a*=a+\frac{\left|e\right|E}{\omega_{0}^{2}}$$

$$(2)\;q<0;\;U_{2}=\frac{\omega_{0}^{2}}{2}{\left(q+a\right)}^{2}-\left|e\right|Eq=\frac{\omega_{0}^{2}}{2}{\left(q+a**\right)}^{2}+a\left|e\right|Eq-\frac{\left|e\right|E}{2\omega_{0}^{2}},\;\mathrm{where}\;a**=a-\frac{\left|e\right|E}{\omega_{0}^{2}}$$

Then the renormalized potential takes the form:

(A2) $$U=\left[\frac{\omega_{0}^{2}}{2}{\left(q-a*\right)}^{2}-a\left|e\right|Eq\right]\theta (q)+\left[\frac{\omega_{0}^{2}}{2}{\left(q+a**\right)}^{2}+a\left|e\right|Eq\right]\theta (-q).$$

The values of the shifted minima (Figure A1) are equal to:

$$U_{1}(a*)=-a\left|e\right|E-\frac{{\left|e\right|}^{2}E^{2}}{2\omega_{0}^{2}},\;U_{2}(-a**)=a\left|e\right|E-\frac{{\left|e\right|}^{2}E^{2}}{2\omega_{0}^{2}},$$

and the shift of the minima turns out to be proportional to the field strength:

(A3) $$\left|\Delta U\right|=U_{2}-U_{1}=2a\left|e\right|E\Rightarrow \left|\Delta U\right|\sim E$$

In this case, the shifts of the minima turn out to be the same in magnitude:

$$\Delta q_{1}=a*-a=\frac{\left|e\right|E}{\omega_{0}^{2}},\;\Delta q_{2}=-a**+a=\frac{\left|e\right|E}{\omega_{0}^{2}}.$$

In the considered model, the top of the potential barrier is fixed:

$$U(0)=\frac{\omega_{0}^{2}a^{2}}{2},$$

but there is a corresponding shift in the value of the left minimum, and as a consequence, the barrier effectively decreases:

(A4) $$\Delta U_{2}=U(0)-U_{2}(-a**)=\frac{\omega_{0}^{2}a^{2}}{2}-a\left|e\right|E+\frac{{\left|e\right|}^{2}E^{2}}{2\omega_{0}^{2}}=\frac{{\left|e\right|}^{2}}{2\omega_{0}^{2}}{\left(E-\frac{a}{\left|e\right|}\omega_{0}^{2}\right)}^{2}$$

Since the subsequent consideration assumes the usage of the semiclassical instanton approximation when calculating the tunneling probability in a double-well oscillatory potential, we will assume that the barrier value cannot be too small in comparison with the sub-barrier transfer length; therefore, there is a natural limitation on the magnitude of the electric field strength:

(A5) $$E<<\frac{a}{\left|e\right|}\omega_{0}^{2}\Rightarrow E<<\frac{ma\omega_{0}^{2}}{\left|e\right|}.$$

In the case when the initial potential turns out to be asymmetric, the situation is similar with a correction for the initial asymmetry parameter (Figure A2).

The initial potential asymmetry is determined by the parameter ΔI:

(A6) $$\tilde{U}(q)=\frac{\omega_{0}^{2}}{2}{\left(q-b\right)}^{2}\theta (q)+\left[\frac{\omega_{0}^{2}}{2}{\left(q+a\right)}^{2}-\Delta I\right]\theta (-q)-\left|e\right|Eq.$$

where $\Delta I=\frac{\omega_{0}^{2}}{2}(a^{2}-b^{2})$; in this case, the displacements of the minima in the external electric field are determined by the parameters:

$$(1)\;q>0;\;{\tilde{U}}_{1}=\frac{\omega_{0}^{2}}{2}{\left(q-b\right)}^{2}-\left|e\right|Eq=\frac{\omega_{0}^{2}}{2}{\left(q-b*\right)}^{2}-q\left|e\right|E-\frac{{\left|e\right|}^{2}E^{2}}{2\omega_{0}^{2}},\;\mathrm{where}\;b*=b+\frac{\left|e\right|E}{\omega_{0}^{2}}$$

$$(2)\;q<0;\;{\tilde{U}}_{2}=\frac{\omega_{0}^{2}}{2}{\left(q+a\right)}^{2}-\frac{\omega_{0}^{2}}{2}(a^{2}-b^{2})-\left|e\right|Eq=\frac{\omega_{0}^{2}}{2}(q+a*)+a\left|e\right|E-\frac{{\left|e\right|}^{2}E^{2}}{2\omega_{0}^{2}}-\frac{\omega_{0}^{2}}{2}(a^{2}-b^{2}),\;\mathrm{where}\;a*=a-\frac{\left|e\right|E}{\omega_{0}^{2}}.$$

The values of the shifted minima are defined as:

(A7) $${\tilde{U}}_{1}(b*)=-b\left|e\right|E-\frac{{\left|e\right|}^{2}E^{2}}{2\omega_{0}^{2}},\;{\tilde{U}}_{2}(a*)=a\left|e\right|E-\frac{{\left|e\right|}^{2}E^{2}}{2\omega_{0}^{2}}-\frac{\omega_{0}^{2}}{2}(a^{2}-b^{2}).$$

The change in the asymmetry of the potential, as in the previous case, is proportional to the magnitude of the field:

(A8) $$\Delta \tilde{U}={\tilde{U}}_{2}(a*)-{\tilde{U}}_{1}(b*)+\frac{\omega_{0}^{2}}{2}(a^{2}-b^{2})=\left|e\right|E(a+b)\sim E$$

At a certain value of the external field, the initially asymmetric potential with a deeper left-hand well can become symmetric $a_{c}^{*}=b_{c}^{*}$:

$${\tilde{U}}_{1}(b*)={\tilde{U}}_{2}(a*);-b\left|e\right|E-\frac{{\left|e\right|}^{2}E^{2}}{2\omega_{0}^{2}}=a\left|e\right|E-\frac{{\left|e\right|}^{2}E^{2}}{2\omega_{0}^{2}}-\frac{\omega_{0}^{2}}{2}(a^{2}-b^{2}),$$

from here:

(A9) $$E\left|e\right|\left(a+b\right)=\frac{\omega_{0}^{2}}{2}(a-b)(a+b)\;\mathrm{and}\;E_{c}=(a-b)\frac{\omega_{0}^{2}}{2\left|e\right|}$$

In order to use the standard model to determine the probability of dissipative tunneling, we will use the following notation for the renormalized double-well oscillatory potential in an external electric field: $q_{1}=b*=b+\frac{|\;e\;|E}{\omega_{0}^{2}}$, $q_{0}=a*=a-\frac{|\;e\;|E}{\omega_{0}^{2}}$. Then the model renormalized 1D potential can be represented in the standard form (Figure A3).

Taking into account the results obtained earlier in [1,2,3,4,26], the model Hamiltonian of the system can be written as

(A10) $$\overset{⌢}{H}=\frac{p_{1}{}^{2}}{2}+v_{1}(y_{1})+y_{1}\sum_{\alpha =2}^{N}C_{\alpha }y_{\alpha }+\frac{1}{2}\sum_{\alpha =2}^{N}\left(p_{\alpha }{}^{2}+\omega_{\alpha }{}^{2}y_{\alpha }{}^{2}\right),$$

where

(A11) $$v_{1}(y_{1})=\left(\frac{1}{2}\omega_{1}{}^{2}y_{1}{}^{2}+\lambda \;y_{1}\right)\;\theta \left(-\frac{\Delta I}{2\lambda }-y_{1}\right)+\left(\frac{1}{2}\omega_{1}{}^{2}y_{1}{}^{2}-\lambda \;y_{1}-\Delta I\right)\;\theta \left(\frac{\Delta I}{2\lambda }+y_{1}\right)$$

The probability of particle tunneling per unit time can be found in the semiclassical approximation. It is necessary that the de Broglie wavelength of the particle be much less than the characteristic linear scale of the potential. For this, it is quite sufficient that the height of the barrier be much greater than the energy of zero-point vibrations in the well of the initial state [1,26]. In addition to the semiclassical approximation, we must assume that the decay is quasi-stationary (for more details see [1,26]), that is, the width of the level Γ, from which the particle tunnels should be much less than the zero-point vibration energy. For the case of a nonzero temperature, the decay probability per unit time is defined as

(A12) Γ=2TImZReZ.

here Z—is the statistical sum of the system, which, due to the decay, is a complex quantity. A discussion of the justification of this expression for the multidimensional case is given in [1,26]. The appearance of the imaginary part in the statistical sum in the case of a two-well potential due to strong dissipation was considered in [1,26]. With a weak interaction with the oscillators of the medium, coherent tunneling oscillations are possible [1,26] (which are not considered in this work).

The calculation of the probability of 1D—dissipative tunneling (A12) in the form Γ=Bexp(−S) accurate to the preexponential factor B (here S—is the one-instanton semiclassical action) in the one-instanton quasi-classical approximation in the model two-well oscillatory potential is given in [26].

To calculate Γ (A12) in the form Γ=Bexp(−S), following the author’s work [26], it is convenient to represent Z in the form of an integral over trajectories [1,26]:

(A13) $$Z=\prod_{\alpha }\int Dy_{1}\int Dy_{\alpha }\exp\left[-S\left\{y_{1};y_{\alpha }\right\}\right].$$

Since we are not interested in the states of the oscillators in the initial and final states, then along the trajectories $y_{\alpha }(\tau )$ and according to the initial conditions $y_{\alpha }(-\beta /2)=y_{\alpha }(\beta /2)$, (here $\beta \equiv T^{-1}$ or $\beta \equiv \frac{\hbar }{kT}$, where ℏ and k are assumed to be equal to unity), we can integrate. Then the action functional depends only on the trajectory $y_{1}(\tau )$:

(A14) $$S\left\{y_{1}\right\}=\int_{-\beta /2}^{\beta /2}d\tau \left[\frac{1}{2}{\dot{y}}_{1}{}^{2}+v(y_{1})+\frac{1}{2}\int_{-\beta /2}^{\beta /2}d\tau^{\prime }\;K\left(\tau -\tau^{\prime }\right)\;\;y_{1}(\tau )y_{1}(\tau^{\prime })\right],$$

where

(A15) $$v(y_{1})=v_{1}(y_{1})-\frac{1}{2}\sum_{\alpha =2}^{N}\frac{C_{\alpha }{}^{2}}{\omega_{\alpha }{}^{2}}\;\;\;y_{1}{}^{2}.$$

here the potential is renormalized, i.e., the so-called adiabatic potential was introduced (for a discussion of this issue, see [1,26]). The kernel of the integral term in (A14) depends only on the parameters of the oscillators. The Fourier coefficients $\zeta_{n}$ in the expansion of the kernel K(τ) in a Fourier series are defined as

(A16) $$\zeta_{n}=\nu_{n}{}^{2}\sum_{\alpha =2}^{N}\frac{C_{\alpha }{}^{2}}{\omega_{\alpha }{}^{2}\left(\omega_{\alpha }{}^{2}+\nu_{n}{}^{2}\right)}.$$

here $\nu_{n}\equiv 2\pi nT$ is the Matsubara frequency. Now, for the convenience of the calculation, we will shift the coordinate $y_{1}$ so that the maximum of the potential $v(y_{1})$ is at the point q=0, i.e.,

(A17) $$q=y_{1}+\frac{\Delta I}{2\lambda }.$$

Then

(A18) $$v(q)=\frac{1}{2}\omega_{0}{}^{2}{\left(q+q_{0}\right)}^{2}\theta \left(-q\right)+\left[\frac{1}{2}\omega_{0}{}^{2}{\left(q-q_{1}\right)}^{2}-\Delta I\right]\theta \left(q\right),$$

where

(A19) $$\omega_{0}{}^{2}=\omega_{1}{}^{2}-\sum_{\alpha =2}^{N}\frac{C_{\alpha }{}^{2}}{\omega_{\alpha }{}^{2}},\;q_{0}=\frac{\lambda }{\omega_{0}{}^{2}}-\frac{\Delta I}{2\lambda },\;q_{1}=\frac{\lambda }{\omega_{0}{}^{2}}+\frac{\Delta I}{2\lambda }.$$

The type of potential (A18) is shown in Figure A3.

We now turn to the calculation of the semiclassical action in the one-instanton approximation. The partition function Z can be calculated in the semiclassical approximation. It is assumed that the main contribution to the action S{q} is made by the trajectory $q_{B}(\tau )$ (instanton), which minimizes the action functional (A14) and obeys the Euler—Lagrange equation:

(A20) $$-{\ddot{q}}_{B}(\tau )+\frac{\partial \;v(q_{B})}{\partial \;q_{B}}+\int_{-\beta /2}^{\beta /2}d\tau^{\prime }\;\;K\left(\tau -\tau^{\prime }\right)\;\;q_{B}\left(\tau^{\prime }\right)=0.$$

moreover, the trajectory $q_{B}(\tau )$ is sought on the class of periodic functions

(A21) $$q_{B}(\tau )=q_{B}(\tau +\beta ).$$

The type of $q_{B}(\tau )$ is determined from the nature of the motion of the particle in the potential −v(q). The particle begins to move (in the case of zero temperature) at the top of the potential −v(q), i.e., at the point $-q_{0}$, then passes the minimum point ($q_{B}=0$) at the moment of time $\tau =-\tau_{0}$ and reaches the value $q_{B}=q_{0}$ (in the case of a symmetric potential) at the moment of time τ=0. Then the particle repeats the trajectory in reverse order. Such a trajectory is called instanton [1,26]. It is noteworthy that the magnitude of the action on the trajectory $q_{B}(\tau )$ does not depend on the position of the instanton center $\tau_{0}$. Time $\tau_{0}$ is determined from the condition

(A22) $$q_{B}(\tau_{0})=0.$$

The trajectory $q_{B}(\tau )$ is shown in Figure A4, where $\tau_{0}$ is the center of the instanton, Δτ is the width of the instanton.

The introduction of time $\tau_{0}$ greatly facilitates the solution of Equation (A20), since the step functions of the coordinate can be replaced by the corresponding functions of time:

(A23) $$\theta \left(-q_{B}\right)=\theta \left(-\tau_{0}-\tau \right)+\theta \left(\tau -\tau_{0}\right);\;\theta \left(q_{B}\right)=\theta \left(\tau +\tau_{0}\right)-\theta \left(\tau -\tau_{0}\right).$$

We will look for the trajectory $q_{B}(\tau )$ in the form of an expansion in a Fourier series:

(A24) $$q_{B}(\tau )=\beta^{-1}\sum_{n=-\infty }^{\infty }q_{n}\exp\left(i\nu_{n}\tau \right).$$

Expanding also the θ-functions and the kernel K(τ) in Fourier series, we obtain equations for the Fourier coefficients $q_{n}$, which can be solved exactly. Then

(A25) $$q_{B}(\tau )=-q_{0}+\frac{2\left(q_{0}+q_{1}\right)\tau_{0}}{\beta }+\frac{2\omega_{0}{}^{2}\left(q_{1}+q_{0}\right)}{\beta }\sum_{n=1}^{\infty }\frac{\sin\nu_{n}\tau_{0}\;\cdot \;\cos\nu_{n}\tau }{\nu_{n}\left(\nu_{n}{}^{2}+\omega_{0}{}^{2}+\zeta_{n}\right)}$$

where $\nu_{n}=2\pi n/\beta$—Matsubara frequency, and $\zeta_{n}$ is determined from the relation (A16). Next, we substitute (A25) in the expression for the action. Then we find

(A26) $$S_{B}=2\omega_{0}{}^{2}\left(q_{0}+q_{1}\right)q_{0}\tau_{0}-\frac{2\omega_{0}{}^{2}{\left(q_{0}+q_{1}\right)}^{2}\tau_{0}{}^{2}}{\beta }-\frac{4\omega_{0}{}^{4}{\left(q_{0}+q_{1}\right)}^{2}}{\beta }\sum_{n=1}^{\infty }\frac{\sin^{2}\nu_{n}\tau_{0}^{}}{\nu_{n}{}^{2}\left(\nu_{n}{}^{2}+\omega_{0}{}^{2}+\zeta_{n}\right)}$$

Thus, the semiclassical action in the one-instanton approximation is determined analytically. Several special cases for the kernel $\zeta_{n}$ will be analyzed below. Consider a situation where there is no interaction with the environment, i.e., $\zeta_{n}=0$. This case corresponds to one-dimensional tunneling. Then, $\tau_{0}$, determined from Equations (A22) and (A25) is

(A27) $$\tau_{0}=\frac{1}{2\omega_{0}}Arcsh\left[\frac{q_{0}-q_{1}}{q_{0}+q_{1}}\;\;sh\frac{\omega_{0}\beta }{2}\right]+\frac{\beta }{4}.$$

The action is found from expressions (A26) and (A27):

$$S_{B}=\frac{\omega_{0}\left(q_{1}{}^{2}-q_{0}{}^{2}\right)}{2}\;Arcsh\left[\frac{q_{1}-q_{0}}{q_{1}+q_{0}}\;\;sh\frac{\omega_{0}\beta }{2}\right]-\frac{\omega_{0}{}^{2}\left(q_{1}{}^{2}-q_{0}{}^{2}\right)}{4}\beta +$$

(A28) $$+\frac{\omega_{0}{\left(q_{1}+q_{0}\right)}^{2}}{2}\left\{\frac{ch\frac{\omega_{0}\beta }{2}-{\left[1+{\left(\frac{q_{1}-q_{0}}{q_{1}+q_{0}}\right)}^{2}sh^{2}\frac{\omega_{0}\beta }{2}\right]}^{1/2}}{sh\frac{\omega_{0}\beta }{2}}\right\}.$$

In the symmetric case

(A29) $$S_{B}=2\omega_{0}q_{0}{}^{2}\;th\frac{\omega_{0}\beta }{4}.$$

Now let’s move on to calculating the preexponential factor. The preexponential factor is determined by the contribution of trajectories close to the instanton. To do this, we have to expand the action to a quadratic term in deviations $q-q_{B}$ and integrate in the functional space [1,26]. Then the tunneling probability per unit time can be written as

(A30) $$\Gamma =B\exp\left(-S_{B}\right),$$

(A31) $$B={\left[\frac{S_{0}}{2\pi }\;\cdot \;\frac{\det{\left(\frac{\delta^{2}S}{\delta q^{2}}\right)}_{q=-q_{0}}}{\det^{\prime }{\left(\frac{\delta^{2}S}{\delta q^{2}}\right)}_{q=q_{B}(\tau )}}\right]}^{1/2},$$

(A32) $$S_{0}=\int_{-\beta /2}^{\beta /2}{\dot{q}}_{B}{}^{2}(\tau )\;d\tau ,$$

$\det^{\prime }$ means that the zero eigenvalue corresponding to the zero instanton mode is omitted. Note that the derivation of this formula assumes the approximation of an ideal instanton gas [1,26]

(A33) $$\Gamma <<{\left(\Delta \tau \right)}^{-1},$$

where Δτ is the width of the transition from the positive value of the trajectory to the negative one (Figure A4). For the sake of rigor, we note that the trajectory $q_{B}(\tau )$ is the sum of two trajectories—an instanton and an anti-instanton. If $\tau_{0}$ is large, then we can assume that instanton and anti-instanton interact weakly. However, for small $\tau_{0}$, this approximation is incorrect. Therefore, we should talk not about an ideal instanton gas, but about a rarefied gas of instanton—anti-instanton pairs. We define the width ${\left(\Delta \tau \right)}^{-1}$ as

(A34) $${\left(\Delta \tau \right)}^{-1}=\frac{\left|{\dot{q}}_{B}\left(\tau_{0}\right)\right|}{q_{0}}.$$

In (A31) $\det\left(\frac{\delta^{2}S}{\delta q^{2}}\right)$ means the calculation of the product of the eigenvalues of the following equation [1,26]:

(A35) $$\left[-\frac{\partial^{2}}{\partial \tau^{2}}+\frac{\partial^{2}v}{\partial q^{2}}-\lambda \right]\;\;q(\tau )+\int_{-\beta /2}^{\beta /2}d\tau^{\prime }\;K\left(\tau -\tau^{\prime }\right)\;q\left(\tau^{\prime }\right)=0.$$

The second derivative of the potential with respect to the coordinate is taken either at the instanton (semiclassical trajectory) or at the minimum point of the metastable potential. For our potential

(A36) $$\frac{\partial^{2}v}{\partial q^{2}}=\omega_{0}{}^{2}-\omega_{0}{}^{2}\left(q_{0}+q_{1}\right)\;\delta \left(q\right).$$

In doing so, we used the condition

(A37) $$\frac{\omega_{0}{}^{2}\left(q_{1}{}^{2}-q_{0}{}^{2}\right)}{2}=\Delta I.$$

First, we calculate the eigenvalues of Equation (A35) at $q=-q_{0}$. The eigenfunctions, as in the case of instanton, are sought in the class of periodic functions. Expanding the trajectory and the kernel K(τ) in Fourier series, we obtain the eigenvalues $\lambda_{0n}$ of Equation (A35):

(A38) $$\lambda_{0n}=\nu_{n}{}^{2}+\omega_{0}{}^{2}+\zeta_{n}.$$

Next, we find the product of the eigenvalues of Equation (A35) on the instanton trajectory. Now the eigenvalue equation has the form

$$\left[-\frac{\partial^{2}}{\partial \tau^{2}}+\omega_{0}{}^{2}-\lambda \right]\;q(\tau )-\frac{\omega_{0}{}^{2}\left(q_{0}+q_{1}\right)}{\left|{\dot{q}}_{B}(\tau_{0})\right|}\left[\delta \left(\tau +\tau_{0}\right)+\delta \left(\tau -\tau_{0}\right)\right]+$$

(A39) $$+\int_{-\beta /2}^{\beta /2}d\tau^{\prime }\;K\left(\tau -\tau^{\prime }\right)\;q\left(\tau^{\prime }\right)=0.$$

We also seek a solution to this equation in the class of periodic functions. Expanding q(τ), the δ-functions and K(τ) in Fourier series and integrating over τ with a factor $\exp\left(-i\nu_{l}\tau \right)$, we find

(A40) $$\left(\nu_{l}{}^{2}+\omega_{0}{}^{2}+\zeta_{l}-\lambda \right)\;\;q_{l}=\frac{2\omega_{0}{}^{2}\left(q_{0}+q_{1}\right)}{\beta \left|{\dot{q}}_{B}\left(\tau_{0}\right)\right|}\left[A\;\;\;\;\;\;\sin\nu_{l}\tau_{0}+B\;\;\;\;\;\cos\nu_{l}\tau_{0}\right].$$

where

(A41) $$A=\sum_{n=-\infty }^{\infty }q_{n}\;\;\;\sin\nu_{n}\tau_{0},\;B=\sum_{n=-\infty }^{\infty }q_{n}\;\;\;\cos\nu_{n}\tau_{0}.$$

From Equation (A40) you can find $q_{l}$; substitution $q_{l}$ in A and B gives two eigenvalue equations:

(A42) $$\frac{2\omega_{0}{}^{2}\left(q_{0}+q_{1}\right)}{\beta \left|{\dot{q}}_{B}\left(\tau_{0}\right)\right|}\sum_{n=-\infty }^{\infty }\frac{\sin^{2}\nu_{n}\tau_{0}}{\lambda_{0n}-\lambda^{\left(1\right)}}=1,$$

(A43) $$\frac{2\omega_{0}{}^{2}\left(q_{0}+q_{1}\right)}{\beta \left|{\dot{q}}_{B}\left(\tau_{0}\right)\right|}\sum_{n=-\infty }^{\infty }\frac{\cos^{2}\nu_{n}\tau_{0}}{\lambda_{0n}-\lambda^{\left(2\right)}}=1.$$

Taking into account from (A25), that

(A44) $$\left|{\dot{q}}_{B}\left(\tau_{0}\right)\right|=\frac{2\omega_{0}{}^{2}\left(q_{0}+q_{1}\right)}{\beta }\sum_{n=-\infty }^{\infty }\frac{\sin^{2}\nu_{n}\tau_{0}}{\lambda_{0n}},$$

Equations (A42) and (A43) can be reduced to the form

(A45) $$\sum_{n=-\infty }^{\infty }\frac{\sin^{2}\nu_{n}\tau_{0}}{\lambda_{0n}}=\sum_{n=-\infty }^{\infty }\frac{\sin^{2}\nu_{n}\tau_{0}}{\lambda_{0n}-\lambda^{\left(1\right)}},$$

(A46) $$\sum_{n=-\infty }^{\infty }\frac{\sin^{2}\nu_{n}\tau_{0}}{\lambda_{0n}}=\sum_{n=-\infty }^{\infty }\frac{\cos^{2}\nu_{n}\tau_{0}}{\lambda_{0n}-\lambda^{\left(2\right)}}.$$

The first equation contains an eigenvalue λ=0, corresponding to the zero mode; it should be excluded from the product of roots. According to Vieta’s theorem, the product of the roots of the first equation (without λ=0) can be found exactly:

(A47) $$\lambda_{1}{}^{\left(1\right)}\;\cdot \ldots \;\cdot \lambda_{n}{}^{\left(1\right)}\cdot \ldots =\lambda_{01}\cdot \ldots \cdot \lambda_{0n}\cdot \ldots \cdot \sum_{n=-\infty }^{\infty }\frac{\sin^{2}\nu_{n}\tau_{0}}{\lambda_{0n}{}^{2}}{\left(\sum_{n=-\infty }^{\infty }\frac{\sin^{2}\nu_{n}\tau_{0}}{\lambda_{0n}}\right)}^{-1},$$

(A48) $$\lambda_{0}{}^{\left(2\right)}\;\cdot \ldots \;\cdot \lambda_{n}{}^{\left(2\right)}\cdot \ldots =\lambda_{00}\cdot \ldots \cdot \lambda_{0n}\cdot \ldots \cdot \sum_{n=-\infty }^{\infty }\frac{\cos2\nu_{n}\tau_{0}}{\lambda_{0n}{}^{2}}{\left(\sum_{n=-\infty }^{\infty }\frac{\sin^{2}\nu_{n}\tau_{0}}{\lambda_{0n}}\right)}^{-1}.$$

For the final calculation of the preexponential factor, it remains to find the normalization of the zero mode, $S_{0}$, determined by formula (A32). Considering that

(A49) $${\dot{q}}_{B}(\tau )=i\frac{2\omega_{0}{}^{2}{\left(q_{0}+q_{1}\right)}^{}}{\beta }\sum_{n=-\infty }^{\infty }\frac{\sin\nu_{n}\tau_{0}\;e^{i\nu_{n}\tau }}{\lambda_{0n}}.$$

and substituting (A49) into the integrand (A32), we obtain

(A50) $$S_{0}=\frac{4\omega_{0}{}^{4}{\left(q_{0}+q_{1}\right)}^{2}}{\beta }\sum_{n=-\infty }^{\infty }\frac{\sin^{2}\nu_{n}\tau_{0}}{\lambda_{0n}{}^{2}}.$$

Taking into account (A47), (A48) and (A50), we can calculate the pre-exponential factor

(A51) $$B=\frac{2\omega_{0}{}^{2}{\left(q_{0}+q_{1}\right)}^{2}}{{\left(2\pi \beta \right)}^{1/2}}\cdot \sum_{n=-\infty }^{\infty }\frac{\sin^{2}\nu_{n}\tau_{0}}{\lambda_{0n}}{\left(\sum_{n=-\infty }^{\infty }\frac{\cos2\nu_{n}\tau_{0}}{\lambda_{0n}}\right)}^{-1/2}.$$

Thus, the problem of particle tunneling in the model potential (A18) in the one-instanton approximation has been solved analytically. The exponent is determined by the semiclassical action (A26), and the preexponential factor is determined by the expression (A51). In this case, the quasi-stationarity condition (A23) (ideal gas of instanton—anti-instanton pairs) imposes restrictions on the temperature and other parameters of the system.

Let us now investigate the influence of low-frequency oscillations of the medium on the probability of a particle transition in a system with a selected tunneling coordinate. In this case, we will consider several important special cases of the spectrum of vibrations of molecules of the medium in relation to the results obtained at the beginning of this section and relating to the transition probability of a particle interacting with a “thermostat” or the heat-bath in system (A18) with a selected tunneling coordinate.

Consider several special cases for the kernel $\zeta_{n}$.

(a) Let there be no interaction with the environment, i.e., $\zeta_{n}=0$. This situation corresponds to one-dimensional tunneling. Expressions for action in this case (A28), (A29) were obtained above. The pre-exponential factor B (A51) has the form

(A52) $$B=\frac{\omega_{0}{}^{3/2}\left(q_{0}+q_{1}\right)\;\left[ch\frac{\omega_{0}\beta }{2}-{⟮1+{⟮\frac{q_{0}-q_{1}}{q_{0}+q_{1}}⟯}^{2}sh^{2}\frac{\omega_{0}\beta }{2}⟯}^{1/2}\right]}{2\;{\left[\pi \;\;sh\frac{\omega_{0}\beta }{2}\;\;{⟮1+{(\frac{q_{0}-q_{1}}{q_{0}+q_{1}}⟯}^{2}\;sh^{2}\frac{\omega_{0}\beta }{2}⟯}^{1/2}\right]}^{1/2}}.$$

When $\tau_{0}\to 0$, i.e., at $\frac{q_{0}}{q_{1}}<<1$

(A53) $$B=\frac{\omega_{0}{}^{3/2}q_{1}}{\sqrt{\pi }}\;{\left[th\frac{\omega_{0}\beta }{2}\right]}^{3/2}.$$

When $\tau_{0}=\frac{\beta }{4}$, i.e., at $q_{1}=q_{0}$ (symmetrical wells)

(A54) $$B=\frac{2\omega_{0}{}^{3/2}q_{0}}{\sqrt{\pi }}\cdot \frac{sh^{2}⟮\frac{\omega_{0}\beta }{4}⟯}{{\left[sh⟮\frac{\omega_{0}\beta }{2}⟯\right]}^{1/2}}.$$

From (A54) it is seen that at low temperatures

(A55) $$B\approx \frac{\omega_{0}{}^{3/2}}{\sqrt{2\pi }}\;q_{0}\exp⟮\frac{\omega_{0}\beta }{4}⟯,$$

That is the preexponential factor diverges. This fact should not discourage, since at low temperatures for symmetric wells the quasi-stationarity condition (A33) is violated and the semiclassical approximation is unfair, since at zero tunneling energy, there is no wave going to infinity. The theory is limited by the approximation of a free gas of instanton—anti-instanton pairs, i.e., the next inequality is true

(A56) $$\beta \omega_{0}<<\frac{16\;U_{B}}{\omega_{0}},$$

where $U_{B}$—barrier height.

(b) An important case of the spectral phonon density is the ohmic damping approximation, i.e., $\zeta_{n}=\gamma \;\left|\nu_{n}\right|$. This case corresponds to the viscous motion of a particle in the classical limit, where the transition probability per unit time was calculated by Kramers [1,26]. At low temperatures, the series in the expression for the action can be summed up by the Euler-McCloren method [1,26], and

(A57) $$S_{B}=\omega_{0}{}^{2}\left(q_{1}{}^{2}-q_{0}{}^{2}\right)\;\tau_{0}-\frac{{\left(q_{1}+q_{0}\right)}^{2}}{\pi }\cdot \frac{\Lambda_{2}{}^{2}\;\ln\left(\Lambda_{1}/\omega_{0}\right)-\Lambda_{1}{}^{2}\;\ln\left(\Lambda_{2}/\omega_{0}\right)}{\Lambda_{2}-\Lambda_{1}}+\;\frac{\gamma \;{\left(q_{1}+q_{0}\right)}^{2}}{\pi }\;\left(C+2\;\ln2\right)+\frac{\gamma \;{\left(q_{0}+q_{1}\right)}^{2}}{\pi }\;\ln⟮\frac{\omega_{0}\beta }{4\pi }\;\sin^{2}\frac{\tau_{0}\pi }{\beta }⟯+O\left(\beta^{-2}\right),$$

where

(A58) $$\Lambda_{1}=\frac{\gamma }{2}-\sqrt{\frac{\gamma^{2}}{4}-\omega_{0}{}^{2}};\;\Lambda_{2}=\frac{\gamma }{2}+\sqrt{\frac{\gamma^{2}}{4}-\omega_{0}{}^{2}}.$$

here C is Euler’s constant and $\Lambda_{1}\tau_{0}\;,\;\;\Lambda_{2}\tau_{0}\;>>1$.

Of particular interest is the case of a symmetric potential, i.e., when $q_{1}=q_{0}$ and $\tau_{0}=\frac{\beta }{4}$. Then the action diverges at low temperatures:

(A59) $$S_{B}\approx \frac{4q_{0}{}^{2}}{\pi }\cdot \frac{\Lambda_{2}{}^{2}\;\ln\left(\Lambda_{1}/\omega_{0}\right)-\Lambda_{1}{}^{2}\;\ln\left(\Lambda_{2}/\omega_{0}\right)}{\Lambda_{2}-\Lambda_{1}}+\frac{4\gamma \;q_{0}{}^{2}}{\pi }\;\left(C+2\;\ln2\right)+\frac{4\gamma \;q_{0}{}^{2}}{\pi }\;\ln\frac{\beta \;\omega_{0}}{4\pi }.$$

This divergence may indicate the localization of the particle in the well of the metastable state. The phenomenon of particle localization (zero quantum limit in the rate constant) is completely absent in the Einstein model of medium oscillations [1,26] and is a property of a large number of low-frequency medium oscillations responsible for the viscous motion of a tunneling particle. It should be noted that in this case the action can be calculated exactly not only in the low-temperature limit:

(A60) $$S_{B}=\frac{4q_{0}{}^{2}\gamma }{\pi }\;\left(C+2\;\ln2\right)+\frac{4q_{0}{}^{2}}{\pi }\cdot \frac{\Lambda_{2}{}^{2}\;\Psi ⟮\frac{1}{2}+\frac{\Lambda_{1}\beta }{4\pi }⟯-\Lambda_{1}{}^{2}\;\Psi ⟮\frac{1}{2}+\frac{\Lambda_{2}\beta }{4\pi }⟯}{\Lambda_{2}-\Lambda_{1}}.$$

here Ψ is the logarithmic derivative of the Γ (of the Euler function) [1,26]. Note that at γ→0, there is no particle localization.

The pre-exponential factor can be calculated exactly at low temperatures and at any $\tau_{0}$:

(A61) $$B=\frac{2\omega_{0}{}^{2}\;\left(q_{0}+q_{1}\right)\;\ln\left(\Lambda_{2}/\Lambda_{1}\right)\;\sin\left(2\pi \;\tau_{0}/\beta \right)}{\pi^{2}{\left(2\gamma \right)}^{1/2}\;\left(\Lambda_{2}-\Lambda_{1}\right)},$$

where $\Lambda_{1}$ and $\Lambda_{2}$ are determined by expressions (A58). At $\frac{\tau_{0}}{\beta }<<1$

(A62) $$B=2q_{0}\pi \;{\left[\frac{\Lambda_{2}-\Lambda_{1}}{2\;\ln\left(\Lambda_{2}/\Lambda_{1}\right)}\right]}^{3/2}.$$

At $\tau_{0}=\frac{\beta }{4}$

(A63) $$B=\frac{4\omega_{0}{}^{4}q_{0}\beta \;\ln\left(\Lambda_{2}/\Lambda_{1}\right)}{\pi^{2}\left(\Lambda_{2}-\Lambda_{1}\right)\;{\left(2\gamma \right)}^{1/2}}.$$

In this case, the preexponential factor is calculated exactly at an arbitrary temperature; however, due to the cumbersomeness of the expression, we will not present it.

Consider the complete expression for the tunneling rate

(A64) $$\Gamma =B_{0}\;{\left(2\gamma \right)}^{-1/2}\;{⟮\frac{\beta \omega_{0}}{4\pi }⟯}^{1-4\gamma \;q_{0}{}^{2}/\pi }\cdot \exp\left(-S_{B}^{\prime }\right).$$

where

$$B_{0}=\frac{16\omega_{0}{}^{3}q_{0}\;\ln\left(\Lambda_{2}/\Lambda_{1}\right)}{\pi \;\left(\Lambda_{2}-\Lambda_{1}\right)},$$

$$S_{B}^{\prime }=\frac{4q_{0}{}^{2}}{\pi }\cdot \frac{\Lambda_{2}{}^{2}\;\ln\left(\Lambda_{1}/\omega_{0}\right)-\Lambda_{1}{}^{2}\;\ln\left(\Lambda_{2}/\omega_{0}\right)}{\Lambda_{2}-\Lambda_{1}}+\frac{4\gamma \;q_{0}{}^{2}\;\left(C+2\;\ln2\right)}{\pi }.$$

From (A64) it can be seen that, depending on the sign of the exponent at the factor $\beta \;\omega_{0}$, three cases are possible:

(1) Let

(A65) $$\frac{4\gamma \;q_{0}{}^{2}}{\pi }>1,$$

then, with a sufficiently strong damping, the particle is localized in the well of the initial state. This phenomenon is not observed when simulating a chemical reaction with a two-frequency model [1,26]. Localization of a particle means a violation of symmetry for the right and left positions. A similar temperature dependence was obtained for the two-level model [1,26].

(2) For the inverse to (A65) inequality at low temperatures, the quasi-stationarity condition is violated, and the temperature is limited by inequality (A33).

(3) At $\frac{4\gamma \;q_{0}{}^{2}}{\pi }=1$ tunneling rate is independent of temperature.

The condition for the theory applicability for ohmic damping has the form

(A66) $$\frac{4q_{0}\omega_{0}{}^{2}}{\pi \;{\left(2\gamma \right)}^{1/2}}\;{⟮\frac{\beta \;\omega_{0}}{4\pi }⟯}^{1-4\gamma \;q_{0}{}^{2}/\pi }\cdot \exp\left(-S_{B}^{\prime }\right)<<1.$$

(c) For a symmetric form of the potential, the action can be calculated exactly (instanton action (A26) at $\tau_{0}=\frac{\beta }{4}$, $q_{1}=q_{0}$), if we choose $\zeta_{n}$ in the form of the Drude approximation [1,26]:

(A67) $$\zeta_{n}=\frac{\gamma \;\omega_{c}\left|\nu_{n}\right|}{\left|\nu_{n}\right|+\omega_{c}},$$

where $\omega_{c}$—the boundary value of the frequency for the vibrational spectrum. In this case

(A68) $$S_{B}=\frac{4\gamma \;q_{0}{}^{2}\;\left(C+2\;\ln2\right)}{\pi }+\frac{4\omega_{0}{}^{4}q_{0}{}^{2}}{\pi }\cdot [\frac{\left(\omega_{c}-\lambda_{1}\right)\;\Psi ⟮\frac{1}{2}+\frac{\lambda_{1}\beta }{4\pi }⟯}{\lambda_{1}{}^{2}\left(\lambda_{2}-\lambda_{1}\right)\;\left(\lambda_{3}-\lambda_{1}\right)}+\;+\frac{\left(\omega_{c}-\lambda_{2}\right)\;\Psi ⟮\frac{1}{2}+\frac{\lambda_{2}\beta }{4\pi }⟯}{\lambda_{2}{}^{2}\left(\lambda_{1}-\lambda_{2}\right)\;\left(\lambda_{3}-\lambda_{2}\right)}+\frac{\left(\omega_{c}-\lambda_{3}\right)\;\Psi ⟮\frac{1}{2}+\frac{\lambda_{3}\beta }{4\pi }⟯}{\lambda_{3}{}^{2}\left(\lambda_{1}-\lambda_{3}\right)\;\left(\lambda_{2}-\lambda_{3}\right)}],$$

where $\lambda_{1}$, $\lambda_{2}$ and $\lambda_{3}$- absolute values of the roots of the following algebraic equation:

(A69) $$\lambda^{3}-\omega_{c}\lambda^{2}+\left(\omega_{0}{}^{2}+\gamma \;\omega_{c}\right)\;\lambda -\omega_{0}{}^{2}\omega_{c}=0.$$

At low temperatures ($\lambda_{i}\beta >>1$, i=1, 2, 3)

(A70) $$S_{B}\approx \frac{4\gamma \;q_{0}{}^{2}}{\pi }\;\left(C+2\;\ln2\right)+\frac{4\omega_{0}{}^{4}q_{0}{}^{2}}{\pi }\cdot [\frac{\left(\omega_{c}-\lambda_{1}\right)\;\ln\left(\lambda_{1}/\omega_{c}\right)}{\lambda_{1}{}^{2}\left(\lambda_{2}-\lambda_{1}\right)\;\left(\lambda_{3}-\lambda_{1}\right)}+\;+\frac{\left(\omega_{c}-\lambda_{2}\right)\;\ln\left(\lambda_{2}/\omega_{c}\right)}{\lambda_{2}{}^{2}\left(\lambda_{1}-\lambda_{2}\right)\;\left(\lambda_{3}-\lambda_{2}\right)}+\frac{\left(\omega_{c}-\lambda_{3}\right)\;\ln\left(\lambda_{3}/\omega_{c}\right)}{\lambda_{3}{}^{2}\left(\lambda_{1}-\lambda_{3}\right)\;\left(\lambda_{2}-\lambda_{3}\right)}]+\frac{4\gamma \;q_{0}{}^{2}}{\pi }\;\ln\frac{\beta \omega_{c}}{4\pi }+O\left(\beta^{-2}\right).$$

As in the previous case, there is a divergence of the action at low temperatures. This divergence is associated with a linear dependence of $\zeta_{n}\left(\nu_{n}\right)$ at $\nu_{n}\to 0$, i.e., the divergence is determined by small frequencies.

The preexponential factor can also be calculated exactly, but we will not give a complete expression for B, but only note that the temperature dependence of the decay probability completely coincides with the case of ohmic damping. The same dependence on viscosity γ also remains.

(d) Let

(A71) $$\zeta_{n}=\frac{\gamma \;\nu_{n}{}^{2}}{\left|\nu_{n}\right|+\omega_{c}}.$$

Such a model is of interest in the theory of tunneling of color centers [1,26] in solids, provided that the medium affects the particle motion due to coupling with acoustic phonons. In addition, in this case, for a symmetric potential, the action can be calculated exactly. We present only its asymptotic behavior at low temperatures:

(A72) $$S_{B}\approx \frac{4\omega_{0}{}^{4}q_{0}{}^{2}}{\pi }\cdot [\frac{\left(\omega_{c}-\Lambda_{1}\right)\;\ln\left(\Lambda_{1}/\omega_{c}\right)}{\Lambda_{1}{}^{2}\left(\Lambda_{2}-\Lambda_{1}\right)\;\left(\Lambda_{3}-\Lambda_{1}\right)}+\frac{\left(\omega_{c}-\Lambda_{2}\right)\;\ln\left(\Lambda_{2}/\omega_{c}\right)}{\Lambda_{2}{}^{2}\left(\Lambda_{1}-\Lambda_{2}\right)\;\left(\Lambda_{3}-\Lambda_{2}\right)}+\;+\frac{\left(\omega_{c}-\Lambda_{3}\right)\;\ln\left(\Lambda_{3}/\omega_{c}\right)}{\Lambda_{3}{}^{2}\left(\Lambda_{1}-\Lambda_{3}\right)\;\left(\Lambda_{2}-\Lambda_{3}\right)}]+O\left(\beta^{-2}\right),$$

where $\Lambda_{i}$ (i=1, 2, 3)—absolute values of the roots of an algebraic equation

(A73) $$\Lambda^{3}-\left(\omega_{c}+\gamma \right)\;\Lambda^{2}+{\omega_{0}}^{2}\Lambda -\omega_{0}{}^{2}\omega_{c}=0.$$

In this case, no divergence of the action is observed and it takes on a finite value at low temperatures. The form of the spectrum (A71) at small values of $\nu_{n}$ depends in a quadratic manner on $\nu_{n}$, which at low temperatures leads to renormalization of the mass of the tunneling particle, and the problem is thus reduced to one-dimensional tunneling.

The pre-exponential factor is

(A74) $$B\approx B_{0}^{\prime }\gamma^{-1/2}\beta^{2},$$

where $B_{0}^{\prime }$—some factor that does not depend on temperature (we will not present its expression due to its cumbersomeness). From (A74) it follows that the preexponential factor diverges at low temperatures as $\beta^{2}$, however, in contrast to the exponential divergence in the one-dimensional case, here the divergence is weakened to a power law due to the presence of viscous motion of the oscillators of the medium. In fact, the divergence is eliminated by the condition of applicability of inequality (A33), i.e., by approximation of the quasi-stationarity of the kinetic process.

Let us consider (A29) taking into account the interaction with one local phonon mode ($\omega_{L}$). For simplicity, we will assume that this interaction is sufficiently small, i.e., $\frac{C}{\omega_{0}{}^{2}}<<1$ and $\frac{C}{\omega_{L}{}^{2}}<<1$. In this case $D\left(\nu_{n}\right)=-\frac{C^{2}}{\nu_{n}{}^{2}+\omega_{L}{}^{2}}$, where $\nu_{n}=\frac{2\pi \;n}{\beta }$; and $\zeta_{n}=\frac{C^{2}\nu_{n}{}^{2}}{\omega_{L}{}^{2}(\omega_{L}{}^{2}+\nu_{n}{}^{2})}$.

Then we can obtain an expression for the semiclassical action taking into account the local mode of the medium—thermostat (of the heat -bath) in the given dimensionless variables:

(A75) $$\begin{array}{l} \frac{S}{a^{2}\omega }=\frac{1}{2}(b*+1)(3-b*)\tau_{0}^{*/}-\frac{{\left(b*+1\right)}^{2}{\left(\tau_{0}^{*/}\right)}^{2}}{2\beta *}-\frac{{\left(b*+1\right)}^{2}}{2{\tilde{\gamma }}^{/}}\{\frac{\left(1-{\tilde{x}}_{2}^{/}\right)}{\sqrt{{\tilde{x}}_{1}^{/}}}[cth\beta *\sqrt{{\tilde{x}}_{1}^{/}}-\\ \frac{1}{sh\beta *\sqrt{{\tilde{x}}_{1}^{/}}}\left\{ch\left(\left(\beta *-\tau_{0}^{*/}\right)\sqrt{{\tilde{x}}_{1}^{/}}\right)-ch\left(\beta *\sqrt{{\tilde{x}}_{1}^{/}}\right)\right\}+ch\left(\left(\beta *-\tau_{0}^{*/}\right)\sqrt{{\tilde{x}}_{1}^{/}}\right)]-\\ \frac{\left(1-{\tilde{x}}_{1}^{/}\right)}{\sqrt{{\tilde{x}}_{2}^{/}}}\left[cth\beta *\sqrt{{\tilde{x}}_{2}^{/}}-\frac{1}{sh\beta *\sqrt{{\tilde{x}}_{2}^{/}}}\left\{ch\left(\left(\beta *-\tau_{0}^{*/}\right)\sqrt{{\tilde{x}}_{2}^{/}}\right)-ch\left(\beta *\sqrt{{\tilde{x}}_{2}^{/}}\right)\right\}+ch\left(\left(\beta *-\tau_{0}^{*/}\right)\sqrt{{\tilde{x}}_{2}^{/}}\right)\right]\} \end{array}$$

where

$\tau_{0}^{*/}=2\omega \tau *=arcsh\left[\frac{1-b*}{1+b*}sh\beta *\right]+\beta *$, $\beta *=\frac{\beta \omega }{2}$; $b*=\frac{q_{1}}{q_{0}}$—renormalized asymmetry parameter. In addition, the influence of the local mode of the thermostat—medium (of the heat-bath) is taken into account through the following parameters:

$${\tilde{\gamma }}^{/}=\frac{\tilde{\gamma }}{\omega^{2}}=\sqrt{{\left[\frac{\omega_{L}^{2}}{\omega^{2}}+1+\frac{C^{2}}{\omega_{L}^{2}\omega^{2}}\right]}^{2}-4\frac{\omega_{L}^{2}}{\omega^{2}}}=\sqrt{{\left[\omega_{L}*+1+C*\right]}^{2}-4\frac{\omega_{L}^{2}}{\omega^{2}}},\;{\tilde{x}}_{1,2}^{/}=\frac{{\tilde{x}}_{1,2}}{\omega_{0}^{2}}=\frac{\gamma_{1,2}}{\omega_{0}^{2}},$$

where

$$\gamma_{1}=\frac{⟮\omega_{L}^{2}+\omega_{0}^{2}+\frac{C^{2}}{\omega_{L}^{2}}⟯-\sqrt{{⟮\omega_{L}^{2}+\omega_{0}^{2}+\frac{C^{2}}{\omega_{L}^{2}}⟯}^{2}-4\omega_{0}^{2}\omega_{L}^{2}}}{2}>0,$$

$$\gamma_{2}=\frac{⟮\omega_{L}^{2}+\omega_{0}^{2}+\frac{C^{2}}{\omega_{L}^{2}}⟯+\sqrt{{⟮\omega_{L}^{2}+\omega_{0}^{2}+\frac{C^{2}}{\omega_{L}^{2}}⟯}^{2}-4\omega_{0}^{2}\omega_{L}^{2}}}{2}>0.$$

To calculate the preexponential factor taking into account the influence of the local mode $\omega_{L}$ of the medium-thermostat (of the heat-bath), we use the previously obtained general expression [1,26] (A51). In this case, as in the case of calculating the semiclassical instanton (Euclidean) action taking into account the local mode $\omega_{L}$, we use that

$$D(\nu_{n})=-\sum_{\alpha =2}^{N}\frac{C_{\alpha }^{2}}{\omega_{\alpha }^{2}+\nu_{n}^{2}}|{}_{\omega_{L}}\to =-\frac{C^{2}}{\omega_{L}^{2}+\nu_{n}^{2}}=-\frac{C^{2}}{\omega_{L}^{2}}+\xi_{n},$$

where

$$\xi_{n}=\frac{C^{2}}{\omega_{L}^{2}}-\frac{C^{2}}{\omega_{L}^{2}+\nu_{n}^{2}};\;\nu_{n}=\frac{2\pi n}{\beta };\;\beta =\frac{\hbar }{kT},\;\lambda_{0n}=\nu_{n}^{2}+\omega_{0}^{2}+\xi_{n}.$$

Then, to calculate the preexponential factor, we will take into account that in the general expression for B (A51)

$$B=\frac{2\omega_{0}^{2}{\left(a+b\right)}^{2}}{{\left(2\pi \beta \right)}^{1/2}}\cdot \frac{\sum_{n=-\infty }^{\infty }\frac{\sin^{2}\nu_{n}\tau_{0}}{\lambda_{0n}}}{{\left[\sum_{n=-\infty }^{\infty }\frac{\cos2\nu_{n}\tau_{0}}{\lambda_{0n}}\right]}^{1/2}}.$$

And the following transformation of expressions occurs:

$$\sum_{n=-\infty }^{\infty }\frac{\sin^{2}\nu_{n}\tau_{0}}{\nu_{n}^{2}+\omega_{0}^{2}+\frac{C^{2}}{\omega_{L}^{2}}-\frac{C^{2}}{\omega_{L}^{2}+\nu_{n}^{2}}}=\sum_{n=-\infty }^{\infty }\frac{1/2(1-\cos2\nu_{n}\tau_{0})}{\nu_{n}^{2}+\omega_{0}^{2}+\frac{C^{2}}{\omega_{L}^{2}}-\frac{C^{2}}{\omega_{L}^{2}+\nu_{n}^{2}}}=$$

$$=\sum_{n=-\infty }^{\infty }\frac{1}{2}\frac{\left(\nu_{n}^{2}+\omega_{L}^{2})(1-\cos2\nu_{n}\tau_{0}\right)}{\nu_{n}^{2}(\nu_{n}^{2}+\omega_{L}^{2})+\omega_{0}^{2}(\nu_{n}^{2}+\omega_{L}^{2})+\frac{C^{2}}{\omega_{L}^{2}}(\nu_{n}^{2}+\omega_{L}^{2})-C^{2}}=$$

$$=\frac{1}{2}\sum_{n=-\infty }^{\infty }\frac{\left(\alpha +\omega_{L}^{2})(1-\cos2\nu_{n}\tau_{0}\right)}{\alpha^{2}+\alpha \left(\omega_{L}^{2}+\omega_{0}^{2}+\frac{C^{2}}{\omega_{L}^{2}}\right)+\omega_{0}^{2}\omega_{L}^{2}}=\frac{\left(\alpha +\omega_{L}^{2})(1-\cos2\nu_{n}\tau_{0}\right)}{\left(\alpha -\alpha_{1})(\alpha -\alpha_{2}\right)}$$

where

$$\alpha =\nu_{n}^{2}=\frac{4\pi^{2}n^{2}}{\beta^{2}},\;\alpha_{1,2}=\frac{-\left(\omega_{0}^{2}+\omega_{L}^{2}+\frac{C^{2}}{\omega_{L}^{2}}\right)\pm \sqrt{{\left(\omega_{0}^{2}+\omega_{L}^{2}+\frac{C^{2}}{\omega_{L}^{2}}\right)}^{2}-4\omega_{0}^{2}\omega_{L}^{2}}}{2}.$$

The expression in the denominator (A51) is converted to the form:

$${\left[\sum_{n=-\infty }^{\infty }\frac{\cos2\nu_{n}\tau_{0}}{\nu_{n}^{2}+\omega_{0}^{2}+\frac{C^{2}}{\omega_{L}^{2}}-\frac{C^{2}}{\omega_{L}^{2}+\nu_{n}^{2}}}\right]}^{1/2};\;\nu_{n}=\frac{2\pi n}{\beta },\;\alpha =\nu_{n}^{2};$$

$$\sum_{n=-\infty }^{\infty }\frac{\cos2\nu_{n}\tau_{0}(\nu_{n}^{2}+\omega_{L}^{2})}{\alpha (\alpha +\omega_{L}^{2})+\omega_{0}^{2}(\alpha +\omega_{L}^{2})+\frac{C^{2}}{\omega_{L}^{2}}(\alpha +\omega_{L}^{2})-C^{2}}=\frac{1}{2}\sum_{n=-\infty }^{\infty }\frac{\cos\frac{4\pi \tau_{0}}{\beta }n(\alpha +\omega_{L}^{2})}{\alpha^{2}+\alpha \left(\omega_{L}^{2}+\omega_{0}^{2}+\frac{C^{2}}{\omega_{L}^{2}}\right)+\omega_{0}^{2}\omega_{L}^{2}}=$$

$$=\frac{1}{2}\sum_{n=-\infty }^{\infty }\frac{\cos\frac{4\pi \tau_{0}}{\beta }n(\alpha +\omega_{L}^{2})}{\left(\alpha -\alpha_{1})(\alpha -\alpha_{2}\right)}$$

where $\alpha_{1,2}$ have been defined above.

Introducing, as in the case of the action calculation, taking into account the local mode of the medium-thermostat (of the heat-bath), the coefficients:

$$\gamma_{1}=-\alpha_{1}=\frac{⟮\omega_{L}^{2}+\omega_{0}^{2}+\frac{C^{2}}{\omega_{L}^{2}}⟯-\sqrt{{⟮\omega_{L}^{2}+\omega_{0}^{2}+\frac{C^{2}}{\omega_{L}^{2}}⟯}^{2}-4\omega_{0}^{2}\omega_{L}^{2}}}{2}>0,$$

$$\gamma_{2}=-\alpha_{2}=\frac{⟮\omega_{L}^{2}+\omega_{0}^{2}+\frac{C^{2}}{\omega_{L}^{2}}⟯+\sqrt{{⟮\omega_{L}^{2}+\omega_{0}^{2}+\frac{C^{2}}{\omega_{L}^{2}}⟯}^{2}-4\omega_{0}^{2}\omega_{L}^{2}}}{2}>0,$$

and also, considering that

$$\frac{\alpha +\omega_{L}^{2}}{\left(\alpha -\alpha_{1})(\alpha -\alpha_{2}\right)}=\frac{1}{2}⟮\frac{B}{\nu_{n}^{2}-\alpha_{1}}+\frac{D}{\nu_{n}^{2}-\alpha_{2}}⟯,$$

where

$$A=-\frac{\left(\omega_{L}^{2}+\alpha_{1}\right)}{\alpha_{2}-\alpha_{1}}=-\frac{\left(\omega_{L}^{2}-\gamma_{1}\right)}{\gamma_{1}-\gamma_{2}};\;D=\frac{\omega_{L}^{2}+\alpha_{2}}{\alpha_{2}-\alpha_{1}}=\frac{\omega_{L}^{2}-\gamma_{2}}{\gamma_{1}-\gamma_{2}},$$

we obtain the final analytical expression for the preexponent taking into account the influence of the local mode of the medium-thermostat (of the heat-bath):

(A76) B˜*=2ω02(a+b)2(2πβ)1/2{A2γ1[γ1βcth⟮γ1β2⟯−1]+D2γ2[γ2βcth⟮γ2β2⟯−1]{A2[β2γ1ch⟮γ1⟮β2−2τ0⟯⟯shγ1β2−1γ1]+D2[β2γ2ch⟮γ2⟮β2−2τ0⟯⟯shγ2β2−1γ2]}12+A2⟮1γ1−β2γ1ch[γ1⟮β2−2τ0⟯]shγ1β2⟯+D2⟮1γ2−β2γ2ch[γ2(β2−2τ0⟯]shγ2β2⟯{A2[β2γ1ch⟮γ1⟮β2−2τ0⟯⟯shγ1β2−1γ1]+D2[β2γ2ch⟮γ2⟮β2−2τ0⟯⟯shγ2β2−1γ2]}12}

For subsequent numerical estimates, we use the introduction of dimensionless parameters $\omega_{L}^{*}={⟮\frac{\omega_{L}}{\omega_{0}}⟯}^{2}$, $C*={⟮\frac{C}{\omega_{L}\omega_{0}}⟯}^{2}$,

$$\gamma_{1,2}=\omega_{0}^{2}\left[\frac{⟮\frac{\omega_{L}^{2}}{\omega_{0}^{2}}+1+\frac{C^{2}}{\omega_{L}^{2}\omega_{0}^{2}}⟯\mp \sqrt{{⟮\frac{\omega_{L}^{2}}{\omega_{0}^{2}}+1+\frac{C^{2}}{\omega_{L}^{2}\omega_{0}^{2}}⟯}^{2}-\frac{4\omega_{L}^{2}}{\omega_{0}^{2}}}}{2}\right]$$

$$=\omega_{0}^{2}\left[\frac{(\omega_{L}*+1+C*)\mp \sqrt{(\omega_{L}*+1+C*)-4\omega_{L}*}}{2}\right]$$

$$\sqrt{\gamma_{1,2}}=\omega_{0}\sqrt{\frac{⟮\frac{\omega_{L}^{2}}{\omega_{0}^{2}}+\frac{C^{2}}{\omega_{L}^{2}\omega_{0}^{2}}+1⟯\mp \sqrt{{⟮\frac{\omega_{L}^{2}}{\omega_{0}^{2}}+\frac{C^{2}}{\omega_{L}^{2}\omega_{0}^{2}}+1⟯}^{2}-\frac{4\omega_{L}^{2}}{\omega_{0}^{2}}}}{2}};$$

$$\begin{array}{l} =\omega_{0}\sqrt{\frac{(\omega_{L}*+1+C*)\mp \sqrt{(\omega_{L}*+1+C*)-4\omega_{L}*}}{2}} \end{array}$$

Wherein:

$$A=-\frac{\left(\omega_{L}^{2}-\gamma_{1}\right)}{\gamma_{1}-\gamma_{2}}=\frac{\omega_{L}*-\frac{1}{2}\left[(\omega_{L}*+1+C*)-\sqrt{(\omega_{L}*+1+C*)-4\omega_{L}*}\right]}{2\sqrt{(\omega_{L}*+1+C*)-4\omega_{L}*}}$$

$$D=\frac{\left(\omega_{L}^{2}-\gamma_{2}\right)}{\gamma_{1}-\gamma_{2}}=\frac{\omega_{L}*-\frac{1}{2}\left[(\omega_{L}*+1+C*)+\sqrt{(\omega_{L}*+1+C*)-4\omega_{L}*}\right]}{2\sqrt{(\omega_{L}*+1+C*)-4\omega_{L}*}}$$

As earlier:

$$\tau *=\frac{\tau_{1}+\tau_{2}}{2}=\frac{1}{2\omega }\tau_{0}=\frac{1}{2\omega }\left[arcsh\left[\frac{1-b*}{1+b*}sh\frac{\beta \omega }{2}\right]+\frac{\beta }{4}\right]$$

Condition (A33), which limits the applicability of the considered approximation, for studying tunneling in semiconductor quantum dots gives the following estimates. The applicability of the semiclassical instanton approximation [1,2,3,4,26] in the study of the temperature dependence of the tunneling probability Γ for QDs based on InSb can be estimated in the semiclassical approximation by comparing the characteristic size of the system with the de Broglie wavelength of the tunneling particle, or in the framework of the rarefied gas approximation pairs “instanton—anti-instanton” [1,2,3,4,26].

$$\left\{ \begin{array}{l} R>>\frac{\hbar }{(2-\sqrt{3})\sqrt{2m^{*}U_{0}}}\\ R>>\frac{\hbar }{\sqrt{8m^{*}k_{B}T}} \end{array}\right.\;,$$

where $U_{0}$—barrier height, $m^{*}$—effective mass of a tunneling electron.

The first inequality compares the QD radius R with the de Broglie wavelength of the tunneling particle; the second formula demonstrates the applicability of the approximation of a rarefied gas of “instanton–anti-instanton” pairs [1,26]. Both inequalities are fulfilled simultaneously at T≥50 K and $U_{0}\approx 0.2\;эB$, which may correspond to QD based on InSb. As shown in the work [27], the suppression of Coulomb effects can occur if the starting energy of the particle in the QD significantly exceeds the energy of the Coulomb repulsion: $U_{0}>>\frac{e^{2}}{q_{0}+q_{1}}$. Supplementing this condition with a limitation on the magnitude of the electric field strength $E<<\frac{U_{0}}{\left|e\right|(q_{0}+q_{1})}$ for QDs made of InSb, we can obtain the following value of the strength: $E<<3\cdot 10^{6}\;V/m$.

As a result, an expression for the probability of one-dimensional tunneling transport is found analytically [1,26]:

(A77) Γ=Bexp(−S).

The inset to Figure 3a of the text of the article shows a graph containing a single peak for the case of a symmetric double-well potential at a certain value of the external electric field strength, obtained as the field dependence of the probability of dissipative tunneling (A77). This peak, obtained theoretically, qualitatively coincides with individual experimental tunneling I–V characteristics for single QDs in the combined AFM/STM system [1].
